# Recovery Colleges or Something Different? The Development and Evaluation of a Reflection Tool for Recovery Colleges in the Netherlands

**DOI:** 10.1007/s10597-025-01517-1

**Published:** 2025-10-29

**Authors:** Marloes van Wezel, Christien Muusse, Jenny Boumans, Dike van de Mheen, Hans Kroon

**Affiliations:** 1 Tranzo Scientific Center for Care and Wellbeing, School of Social and Behavioural Sciences, Tilburg University, Tilburg, the Netherlands; 2 Department of Reintegration and Community Care, Trimbos Institute, Utrecht, the Netherlands

**Keywords:** Recovery colleges, Fidelity measure, Peer support, Reflection, Co-creation

## Abstract

Recovery Colleges (RCs) are increasingly implemented worldwide, with a fidelity measure recently developed in the United Kingdom (UK). However, RCs may vary based on their operating contexts. In the Netherlands, a key deviation is that RCs are often peer-run rather than co-produced with mental healthcare providers, as outlined in the fidelity measure. This study assessed the measure’s suitability in the Dutch context, leading to the development of a reflection tool (Phase 1) which was subsequently evaluated (Phase 2). The development phase involved focus groups with peer facilitators and coordinators from 16 RCs (*N =* 29) to capture critical elements of Dutch RCs. The tool was piloted (*N =* 5) and evaluated more broadly (*N =* 24). The tool provided valuable insights, highlighting shared values among RCs internationally and conceptual differences between Dutch and UK models. The findings raised questions about the boundaries of fidelity in co-created contexts such as RCs.

**Supplementary Information:**

The online version contains supplementary material available at 10.1007/s10597-025-01517-1.

## Introduction

Recovery Colleges (RCs) facilitate empowering recovery journeys of individuals with diverse mental health experiences seeking to improve their mental wellbeing or navigate their personal recovery process (Perkins et al., 2018; Whitley et al., 2019). Rather than a therapeutical model, RCs adopt an educational model where collaborative learning with peers is a key aspect (Jay et al., 2017; Toney et al., 2019). RCs make way for individuals to (re)gain agency about what they need and want to live a meaningful life, despite their vulnerabilities, in a peer supported learning environment. Inspired by American ‘Recovery Learning Centers’ (Perkins et al., 2018; Recovery College YVR, n.d.) the RC movement rapidly spread across the globe after the first RCs opened their doors in the United Kingdom (UK) in 2009. Recovery-oriented care and related interventions, including RCs, are increasingly promoted (e.g., Slade et al., 2014), and initial research into RC effectiveness suggests promising outcomes (Crowther et al., 2019; Lin et al., 2023; Thériault et al., 2020). Several (qualitative) evaluations have related RC attendance to positive outcomes in both personal recovery (Ebrahim et al., 2018; Meddings et al., 2015; Thompson et al., 2021; Wilson et al., 2019) and societal recovery (Beckers & Koopmans, 2025; Meddings et al., 2014; Sutton et al., 2019). As a result, the implementation of RCs continues to gain momentum (Hopkins et al., 2022; van Os et al., 2019). A recent international inventory included 221 RCs across 28 countries and five continents (Hayes et al., 2023b).

Scholars, often in co-creation with peers, have attempted to grasp the essence of RCs in several forms. This includes defining core features (Perkins et al., 2012, 2018), developing a values and ethics checklist (SAMHSA, 2014), or exploring mechanisms of change and action (Thompson et al., 2021; Toney et al., 2018). Central elements described are for example co-production, peer support and empowerment. Toney et al. (2019) were the first to develop a fidelity measure for Recovery Colleges, which has been tested recently within the UK (Hayes et al., 2023a) and internationally (Hayes et al., 2023b). A defined fidelity measure is considered important to ensure that ‘interventions’ are implemented as intended, allowing researchers and practitioners to accurately assess its effectiveness, minimize implementation variability and ensuring the reliability and generalizability of outcomes (Mowbray et al., 2003).

In the Netherlands, RCs have also gained a foothold in the care and support domain, though drafting accurate prevalence rates is challenging for two reasons. First, in the Netherlands, there is no consensus in using the term ‘Recovery Colleges’. The term ‘self-direction and recovery initiatives’ is used as an overarching term that includes RCs and also initiatives that do not call themselves RC, but seem to have a close resemblance^1^. Second, most (if not all) Dutch RCs are not in line with the RC model from the UK. This may explain why the international inventory by Hayes and colleagues (2023) only reported 2 RCs being active in the Netherlands, while in 2024, MIND, a consumer and family advocacy organization, reported 192 self-direction and recovery initiative locations (MIND, 2024).

To be more specific, many Dutch RCs adopt a 100% peer support approach, without co-production and collaboration with mental healthcare providers (Dutch Association for Self-Direction and Recovery, 2022). This contrasts with RCs from the UK, where coproduction with healthcare providers is a key element (Morgan et al., 2019; Toney et al., 2019). A possible explanation for this deviation is the strong user emancipatory movement that rapidly gained momentum in the Netherlands from 1970 onwards. Central to this movement was promotion of patient advocacy, rights and empowerment, allowing individuals to make own decisions about their recovery journey (criticizing the paternalistic and depending-inducing nature of the psychiatry at that time: Boevink, 2017; Hunsche & van Andel, 2008). Dutch RCs are rooted in this movement, aiming to facilitate an alternative space to regular services. This does not mean that they are isolated from regular services: some RCs are organized as independent foundations, but most have been developed under the wings of a host organization in mental health care or supported housing. In the Netherlands, RCs are increasingly considered as complementary to community centers, social services and health care. In that light, Dutch RCs may be seen as a parallel movement *besides* regular mental healthcare services, while RCs following the UK model are more occupied with promoting culture change *within* mental healthcare services (Perkins et al., 2018; King & Meddings, 2019).

Following from this, we aim to answer the following research questions:

- RQ 1: What are the core elements of RCs in the Netherlands?
- RQ 2: In what way is the model of Dutch RCs different from what is specified in the UK Fidelity Measure?
- RQ 3: What is the fidelity of Dutch RCs?
- RQ 4: Do Dutch initiatives labeled as ‘RC’ conceptually differ from peer-supported recovery initiatives that adopt other labels?

This study is part of a large research project investigating the meaning and effectiveness of RCs in the Netherlands (van Wezel et al., 2023). In short, we developed a Dutch Reflection Tool (instead of the originally planned Dutch Fidelity Measure; phase 1) and tested this tool in a pilot and on a larger scale (phase 2). The methods and results are described for these phases subsequently.

## PHASE 1 – Development of the Tool

### Methods

#### Design

This study used a qualitative, participatory research design to identify key elements of RCs and collaboratively develop a description of Dutch RCs. Focus groups were conducted to encourage collective reflection and dialogue among stakeholders from the field. Experiential co-researchers (partakers of Enik RC, see van Wezel et al., 2023) played an integral role in co-designing the study, facilitating focus groups, and contributing to data analysis, ensuring that the process was grounded in practical and experiential knowledge (Slattery et al., 2020; Waddingham, 2015; Walmsley et al., 2018).

#### Recruitment

**Fig. 1:**
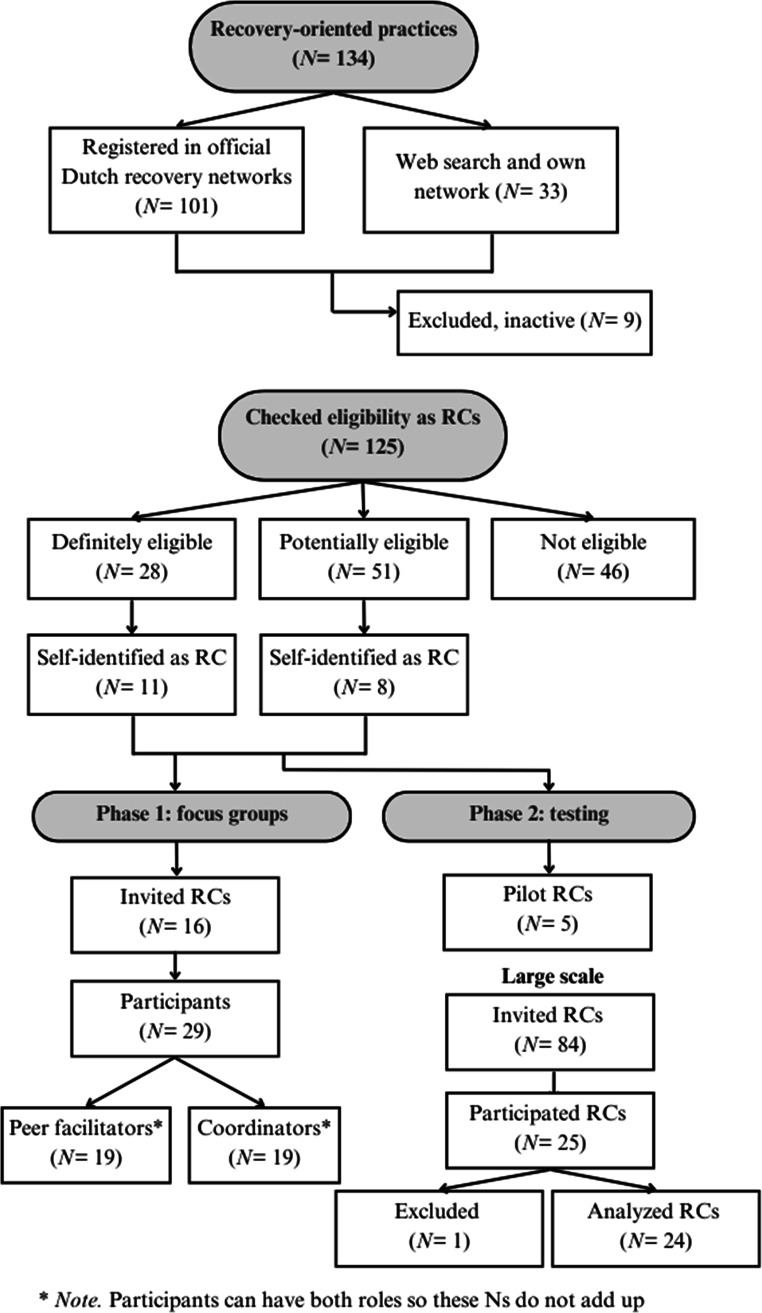
Flow chart of enrollment procedure for phases 1 and 2

Given the lack of a comprehensive overview of Dutch RCs and inconsistent terminology, identifying suitable initiatives posed challenges (see Fig. 1 for enrollment details). First, a general overview of Dutch ‘recovery-oriented practices’ was compiled. Initiatives should be physically located in the Netherlands (thereby excluding fully online services) and operate within mental health care or community and social services (thereby excluding commercial coaching). Our search, conducted between January and March 2023, utilized Dutch recovery networks (MIND Atlas, the Dutch Association for Self-direction and Recovery, psychosenet.nl, socialekaartnederland.nl) complemented by web searches and network input. This yielded a list of 134 initiatives, with 9 found inactive during phase 1 recruitment.

This overview was narrowed down to initiatives that met the pre-defined eligibility criteria (van Wezel et al., 2023). Among the 125 active initiatives, 28 were definitely eligible as RC, 46 were definitely not eligible, and 51 were challenging to assess due to limited information or interpretive eligibility criteria. The RCs that were invited to participate in a focus group were carefully selected from the potential and definitely eligible pool (*N =* 79), to encompass all key variations (e.g., longevity and geographical density, see van Wezel et al., 2023).

#### Participants

A total of 29 participants, representing 16 Dutch RCs, took part in the focus groups, consisting of 10 RC coordinators, 10 peer facilitators and 9 who fulfilled both roles. On average, participants had been involved with the RC for 4.93 years (*SD =* 3.01). In addition to the focus group participants, a team of experts (e.g., from MIND and the Dutch Association for Self-Direction and Recovery) were consulted.

#### Materials

The RECOLLECT Fidelity Measure (Toney et al., 2019) was translated to Dutch^2^ as a starting point for discussion in the focus groups and provided to participants beforehand (Appendix A). This fidelity measure distinguishes 7 nonmodifiable components (rated on an ordinal scale from 0 to 2) and 5 modifiable components (rated on a binary scale, distinguishing Type I vs. Type 2) in a structured checklist. The components are operationalizations of RC principles as defined by Perkins and Repper (2017). Nonmodifiable components are: Valuing Equality, Learning, Tailored to the Student, Coproduction of the Recovery College, Social Connectedness, Community Focus, and Commitment to Recovery. Modifiable components are: Available to All, Location, Distinctiveness of Course Content, Strengths Based, and Progressive. Scores of the nonmodifiable components are used to compute a fidelity score, with higher scores representing higher fidelity.

#### Procedure

Coordinators and peer facilitators participated in 2-hour online focus groups with maximally 8 participants, after providing informed consent. All focus groups started with a discussion on the characteristics of Dutch RCs using a word cloud. Then, three to six items from the translated Fidelity Measure were discussed in-depth to establish what was (not) in line with Dutch RCs. Each item was discussed at least twice, and participants could provide additional feedback via e-mail. The focus groups were moderated by two academic researchers and one experiential co-researcher. All meetings were audio-recorded, transcribed verbatim and pseudonymized. Participants also completed a short online survey in Qualtrics about their demographics and descriptives of the RC.

#### Analysis

Input from the focus groups and various expert meetings was analyzed using reflexive thematic analysis (Braun & Clarke, 2013; Braun et al., 2022) in co-creation with experiential co-researchers and a team of experts (see van Wezel et al., 2023). Using MAXQDA, the first author deductively coded RECOLLECT fidelity components and used inductive coding to identify context-specific elements. Together with the experiential co-researchers, these were merged into overarching themes, outlining Dutch RC characteristics in relation to the existing fidelity measure (Appendix B). The first author refined this overview by comparing it with other (Dutch) RC descriptions (Boertien & Harmsen, 2017; Hellweg, 2020; McGregor et al., 2014; Perkins et al., 2012), and structured it into core values, core tasks, and practical choices. These were supplemented with data-driven descriptions and a scoring scale (where applicable), which were refined in collaboration with the expert team and experiential co-researchers.

Triangulation of focus group data, expert opinions and experiential knowledge from the co-researchers strengthened the validation of the findings and supported an adequate translation of insights into the reflection tool. Focus group transcripts were member-checked. In group discussions among experiential- and academic researchers, we reflected on how personal experiences and prior knowledge shaped the tool’s development. Finally, we documented key decisions and methodological considerations directly in our working documents.

### Results

In this section, RQ1 (identifying core elements of Dutch RCs) and RQ2 (comparing Dutch RCs vs. RCs aligning with the UK fidelity measure)^3^ will be addressed.

#### Core Elements

The core elements of Dutch RCs have been summarized into ten core tasks of a Reflection Tool (see section ‘Format Change – Reflection Tool). The focus groups and expert meetings highlighted the importance of equity and reciprocity, experiential knowledge, free space and fostering a learning environment. For example, to emphasize the importance of equity, reciprocity and experiential knowledge, a peer facilitator explained: “Visitors tend to feel more at ease when they know that you also share something about your vulnerability. I believe this is a significant aspect of Recovery Colleges” (Facilitator 1).

Furthermore, so called free space emerged as one of the most essential values at the heart of any RC. This concept stands for (figurative and literal) space enabling individuals to explore their desires and needs, empowering them to take charge of their own recovery journey. One coordinator emphasized the importance of a figurative free space, making way for self-discovery and empowerment:

“A lot of people first enter in the role of patients, because they know that. […] And then the first step is taking the wheel of your own life into your own hands. Making your own decisions, thinking for yourself” (Coordinator 15).

The concept of free space was also emphasized in a literal sense, for example reflected in the absence of predefined pathways, or organizational independence from any facilitating (mental health) organization or funding party. One coordinator compared their experiences of setting up a recovery workshop in a mental health organization with their current work at the RC: “Now I can really make sure that activities and trainings match the needs of participants instead of the needs of a mental health care facility” (Coordinator 1).

#### Highlighted Differences NL vs. UK

The themes outlined in the RECOLLECT Fidelity Measure provided inspiration but required significant content adaptations to align with the Dutch context. The most notable content differences between the UK model and Dutch practice included promoting bottom-up co-creation and offering a diverse range of opportunities that encompass both formal and informal learning.

##### Bottom-Up Co-Creation with Peers 

While in the UK co-production is defined as a collaboration between mental healthcare providers and individuals with lived experience, in the Netherlands a co-creation^4^ approach among peers is embraced. Most focus group participants believed that adopting a 100% peer support philosophy is crucial: “In that, you experience the power of ‘We can do this together’. That’s why at [initiative 6] we say, ‘This is 100% peer support’.” (Coordinator 6). However, this does not necessarily imply that mental healthcare providers^5^ are not welcome: “The healthcare providers would have to enter as participants, and want to participate themselves from experience, as a fellow human being.” (Facilitator 4). This quote illustrates the RC’s values of reciprocity among peers, equity and collaborative learning.

While all recognized the importance of peer support and experiential knowledge, some saw value in co-production bringing together experiential and professional knowledge: “We also […] sometimes invite a family confidant to tell more about, for example, privacy legislation. Because these are topics that family members find interesting and that we just don’t know enough about.” (Facilitator 16). Participants who advocated for possible collaboration with mental healthcare providers also pointed at the stigma towards mental health care and warned that RCs should not fuel that:

“[…] The stigma around mental health care is substantial. And if you let go of that in our living room it is all about ‘Mental health care this and that, it is all worthless’. We try to avoid that and discuss that with each other.” (Coordinator 10a).

##### Formal vs. Informal Learning

The RECOLLECT Fidelity Measure adopts an adult education approach, referring to trainers and students, lesson plans and educational principles to facilitate learning. According to our participants, this terminology was unfamiliar in the Dutch context, where partakers do not like to be called ‘students’: “Then they experience pressure: what do I have to learn? Will I meet those requirements?” (Coordinator 4). Instead, Dutch RCs facilitate a culture of learning, with the training program viewed as one tool among many. Participants for example stressed the importance of making people feel welcome and engaging in low-threshold activities, such as walks or creative exercises. As a facilitator explained:

“Of course, you can take training courses and attend classes to grow. But I do believe that developing yourself—life lessons you gain and your personal growth—that is also learning. […] Does it always have to be educational? No. […] Recovery can happen in other ways as well.” (Facilitator 13).

#### Format Change – Reflection Tool

A traditional fidelity measure was deemed unsuitable for the bottom-up, diverse practices of Dutch RCs, as participants felt it implied a one-size-fits-all approach reminiscent of a quality certification focused on meeting predefined standards. The variety of the Dutch RC practice was evident even in the debate on terminology, as various labels were used: “When you name it you frame it. If it is a movement, flexible, you look from the inside what works. How tight do you want to make it?” (Coordinator 10b). According to the participants, the strength of Dutch RCs lies in their diversity and philosophy of free space and flexibility, suggesting that adherence to every criterion of a ‘model’ is not necessary to embody RC principles. “We can’t pin down how it should look, but we can frame the direction we’re heading. […]. If you claim to operate within the spectrum of recovery initiatives, we can assess the initiative based on those core values.” (Facilitator 5). In turn, participants proposed that the tool’s primary goal should be fostering reflection on implementing core values, rather than evaluating adherence to a specific model.

##### The Developed Reflection Tool

**Fig. 2:**
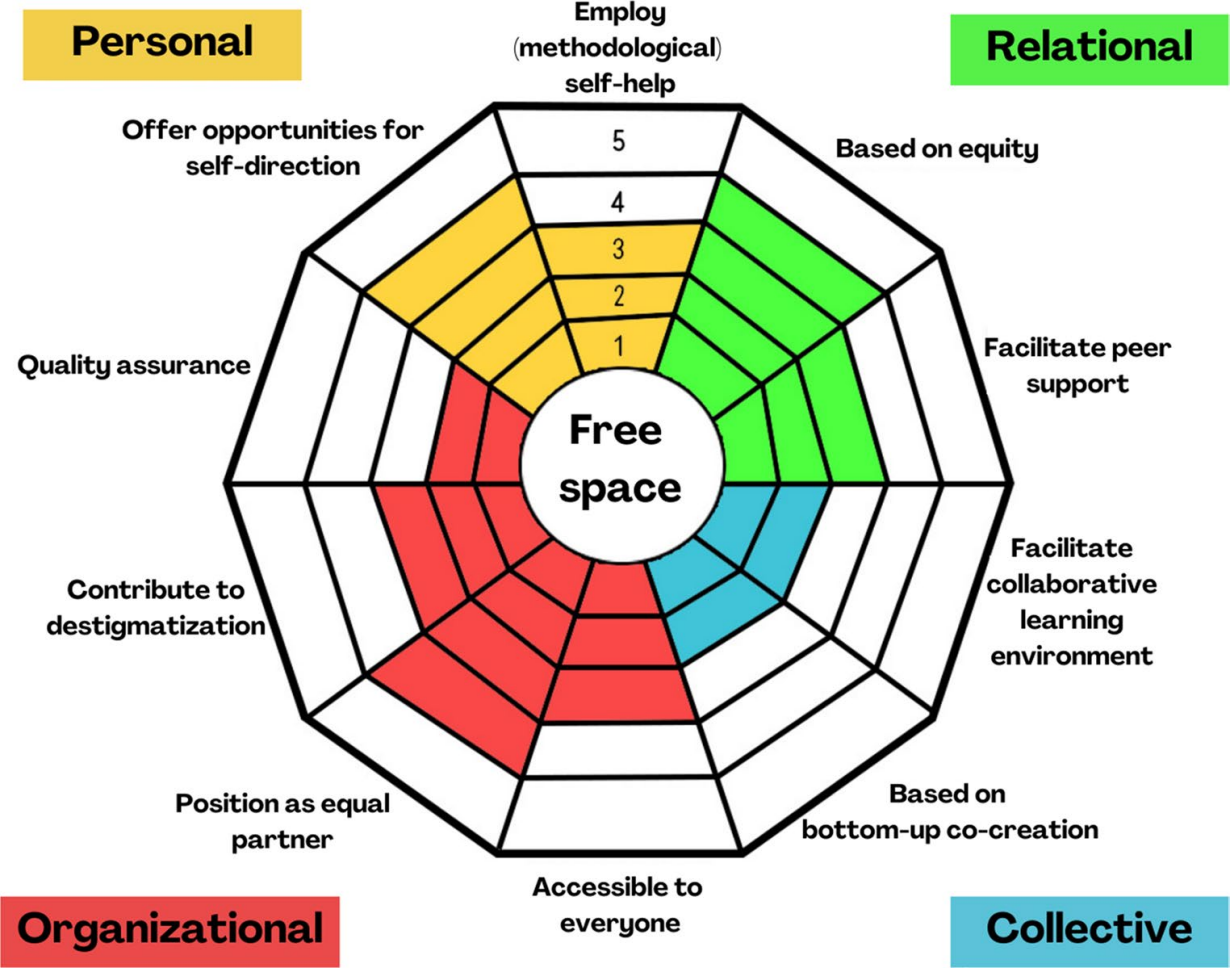
Example scoring of the core tasks of the reflection tool

While the modifiable components of the RECOLLECT fidelity measure (Toney et al., 2019) allowed for flexibility between Type 1 and Type 2 models, we opted to create a new format following from the focus group discussions, letting go of a traditional fidelity measure. The developed Reflection Tool first outlines core values that underpin all RC activities and decisions (such as free space, experiential knowledge and recovery). These abstract values are integral but not directly quantifiable. The tool then defines ten core tasks scored on a scale from 1 (Does not apply at all) to 5 (Applies completely), categorized into four domains (see Fig. 2). The Personal Domain focusses on processes that primarily occur within partakers, the Relational Domain addresses defining features of the interpersonal relationships between partakers, the Collective Domain concerns the broader community and collective identity, and the Organizational Domain relates to operational processes. Sub-questions are provided to prompt reflection and help determine appropriate scores for each task. A template radar tool (as illustrated in Fig. 2) is included with the tool to aid in visualizing strengths and areas for growth and development. After the core tasks, practical choices inspired by Toney et al. (2019) are presented, regarding program scope, co-creation partnerships, and type of location. These practical choices are rated on a 5-point scale, ranging between two extremes: for example, exclusively using RC-owned locations or exclusively using community-shared spaces, with intermediate options that represent varying combinations of both.

The Reflection Tool was tested in a pilot and in a final testing phase, which is reported in the next section.

## PHASE 2 – Testing the Measure

### Methods

#### Recruitment

For the pilot testing, we used a similar strategy as in phase 1 to select a small sub-sample to be invited. For the final testing phase, the tool was distributed on a large scale, including all identified 79 (potentially) eligible active RCs. Additionally, five previously unknown RCs expressed interest in participating after learning about the widespread distribution of the tool and were therefore invited too.

#### Participants

In the pilot, five RCs participated. In the large-scale testing phase, 25 RCs of the 84 invited RCs participated (response rate of 29.8%). Seven were unreachable (and potentially inactive), seven declined, and 41 did not respond. The rest opened the tool but did not complete it or lacked time within the data collection period. One RC was excluded after data collection because it did not meet the eligibility criteria. Of the 24 included RCs, 12 (48%) labeled themselves as Recovery College (or *‘herstelacademie’* in Dutch). Other labels encountered were for example Self-Management Center (*‘zelfregiecentrum’*), Center for Experiential Knowledge (*‘ervaringskenniscentrum’*), Recovery Space (*‘herstelruimte’*) or Recovery Learning Community.

#### Materials

The final tool is based on the data analysis of phase 1, supplemented with extant Dutch characterizations of recovery-oriented initiatives (Dutch Association for Self-Direction and Recovery, 2022; Hellweg, 2020; van Erp et al., 2022; Workgroup accessible support centers, 2023) and of RCs specifically (Boertien & Harmsen, 2017). The format of the tool is described in the results section of phase 1, and a translation of the final tool is provided in Appendix C.

#### Procedure

Prior to the final testing of the tool, we tested the tool in pilots with site visits among five RCs conducted by the first author and an experiential co-researcher. The RC coordinator, oftentimes together with one or more involved peer facilitators, filled out the tool in a thinking-out-loud procedure. Coordinators also filled out an evaluation survey inquiring about face and content validity, comprehensiveness, acceptability and usability (inspired by Toney et al., 2019) and provided organization descriptives. The first author and experiential co-researcher collaboratively reflected on their observations and site visit reports were member-checked.

For the final testing phase the tool was distributed via e-mail to be filled out by as many Dutch RCs as possible. The tool was a self-report tool and should be filled out by the coordinator, ideally in a co-creation session with partakers from all levels of the organization (visitors, students, volunteers, employees, etc.). Several RCs managed to organize such a co-creation session (*N =* 8), of which two were moderated by the first author upon request. In other cases, the coordinator filled out the survey alone (*N =* 11), with one colleague (*N =* 1) or it was unknown due to a missing evaluation survey (*N =* 3) or incomplete data (*N =* 1). Additionally, the same evaluation and descriptive surveys as in the pilot were used.

#### Analysis

The analysis in phase 2 entailed three components: (1) evaluating qualitative and (2) quantitative feedback on the tool, and (3) exploring how the participated RCs scored on the tool. Qualitative feedback was summarized to evaluate the tool’s usability, comprehensiveness and needed adjustments for further improvement. During the two moderated sessions, the first author took notes capturing contextual observations and participant reactions, contributing to our understanding of the applicability of the tool in practice and providing additional suggestions for improvement. Quantitative feedback was analyzed using descriptive statistics. To explore how the participated RCs scored on the tool, descriptive statistics were determined on item-level. Comparative tests (t-test or non-parametric alternatives if needed) were conducted to investigate whether initiatives labeled as RC scored differently on the tool than those who adopted another label. Qualitative data was structured in Excel, quantitative data analysis was conducted using R (version 4.3.2).

### Results

Since the developed tool was not a traditional fidelity measure, the ‘fidelity’ of Dutch RCs (RQ3) cannot be reported. Instead, we provide an overview of how the tool was applied and evaluated. This includes: (1) descriptive information on the participated RCs, (2) a qualitative and quantitative evaluation of the tool, and (3) the final scores (including a comparative analysis of RC-labeled initiatives and peer-supported recovery initiatives labeled differently; RQ4).

#### Descriptive Information

Descriptives were provided by 21 initiatives encompassing a total of 93 locations (Table 1). Standard deviations were large, indicative of the great variety among the participating RCs. For example, the number of unique visitors per year ranged from 12 to 7,800. The average number of volunteers affiliated with participating RCs (*M =* 44.53) was approximately three times higher than the number of employees (*M =* 14.71). The average time in operation was higher than expected (*M =* 10.00, *Median =* 10), but could be explained by the participation of three long-established initiatives (20–26 years), illustrating the historical origins of peer-supported recovery initiatives in the Netherlands. About 38% of the RCs were independent, not affiliated with any host organization.

**Table 1:** Descriptives of participating RCs for phase 2 (Final testing phase, *N* = 21)

	M		SD	Range	
**Time in operation, years**	10.00		7.66	0–26	
Number of students* per year					
Unique (*N =* 20)	936.95	1,694.44			12–7,800
Total	2,232.90	2,991.27			24–10,000
Number of visitors** per year					
Unique	724.12	818.59			30–2,600
Total	2,353.50	2,786.41			150–10,000
**Number of volunteers**	44.53	66.17			2–250
**Number of employees**	14.71	18.31			0–70
**Number of activities per year**					
Unique	18.50	16.21			1–60
Total	100.50	117.68			2–430
**Opening hours per week**	33.05	14.28			6–60
**Unique locations**	4.43	5.15			1–16
	*N*	*%*			
**Organizational affiliation**					
Independent	8	38.1			
Mental health services	7	33.3			
Sheltered living and supported housing	3	14.3			
Other	3	14.3			
**Funding (*N =* 20)**					
Short-term	9	45.0			
Long-term (+ 3 years)	8	40.0			
Combination	1	5.0			
Other	2	10.0			
Type of locations***					
Own locations	7	33.3			
Community-based locations	9	42.9			
Mental healthcare services locations	6	28.6			
Other	6	28.6			
**Founding history**					
Client-driven	7	33.3			
Organization-driven	4	19.1			
Combination	10	47.6			
Additional offerings ****					
Social meeting ground	16	76.2			
Volunteer opportunities	19	90.5			
Creative activities	15	71.4			
Lifestyle activities	10	47.6			
Retreats/overnight stays	3	14.3			
Online	9	42.9			

More tools (*N =* 24) than evaluations (*N =* 21) were completed, so some data is missing

* Students refers to participants of recovery-oriented workshops, trainings or activities

** Visitors refers to people visiting the social meeting ground, not (necessarily) participating in activities

*** RCs can operate across various types of locations

**** Beyond the standard recovery-oriented trainings, workshops and activities

#### Evaluation of the Tool

##### Qualitative Evaluation

Based on pilot input, the tool was revised before the final testing phase. For example, participants noted some redundancy among items, and highlighted that quality assurance was a key theme missing. In response, ‘Quality assurance’ was added as a core task and the overall text was revised.

Qualitative feedback during the final testing phase indicated that the tool made a positive first impression. Participants experienced the tool as helpful to provide insights in both the status quo and opportunities for further development. Some also valued the tool as promoting unification in the field, with one participant stating: “I find it important that we are aligned in terms of policy with other recovery initiatives, rather than ‘just doing something’.” (RC 19). Yet, another warned that the tool should serve a reflective rather than a normative goal:

“As long as it’s used as a tool for self-assessment, where you’re invited to decide for yourself where you’d like to grow, it is a useful tool. However, if the tool is viewed as a standard (where a higher score on all aspects is considered better), it fails to acknowledge the value of initiatives that choose to vary.” (RC 22).

Several areas for improvement were also identified. Despite adjustments after the pilot, the tool was still considered challenging due to its length and complex language. Four coordinators, who completed the tool with RC participants and volunteers, found the language too abstract. They recommended enhancing its usability, shortening it, and clarifying the language to better support collaborative reflection with all partakers.

##### Quantitative Evaluation

Across the pilot and large-scale testing, the tool was rated as comprehensive, valid and usable in practice (Table 2). On average, the tool received favorable ratings on a scale from 1 to 10 (pilot: 7.40, final testing: 7.76).

**Table 2:** Quantitative evaluations of the reflection tool in the pilot and final testing

	Pilot *(N = 5)*		Final testing *(N=21)*	
	*M*	*SD*	*M*	*SD*
**Comprehensiveness of:** The overall tool	4.53	0.38	4.02	0.58
Language use	4.80	0.45	4.10	0.74
Graphics	4.65	0.42	3.99	0.42
**Content validity**	4.53	0.51	3.84	0.72
**Usability**	4.13	0.73	4.13	0.50
**Rating (1–10)**	7.40	1.52	7.76	0.89

Note. Comprehensiveness, content validity and usability was rated on a 5-point Likert scale

#### Final Scores

##### Item Scores and Variances

On average, core tasks that received the lowest scores were ‘Quality assurance’ (*M =* 3.62), ‘Contribute to destigmatization’ (*M =* 3.75), and ‘Position as equal partner’ (*M =* 3.78). Core tasks that received the highest scores were ‘Facilitate peer support’ (*M =* 4.29) and ‘Employ (methodological) self-help’ (*M =* 4.25). The least variance occurred in ‘Accessible to everyone’ (*SD =* 0.58) and the most variance occurred in ‘Position as equal partner (*SD =* 0.88), though differences between items were relatively small.

The practical choices were categorical, with five answer options shifting between two extremes (for example 100% peer-run vs. 100% co-creation with mental healthcare providers). Most variance occurred in the type of location (*M =* 3.17, *SD =* 1.61). Table 3 provides all average scores, and Appendix D provides bar plots for all core tasks (Figure D.1) and practical choices (Figure D.2).

**Table 3:** Average scores on the reflection tool items in the final testing phase (*N* = 24)

Item	M	SD
**Personal domain**		
Offer opportunities for self-direction	4.21	0.59
Employ (methodological) self-help	4.25	0.85
**Relational domain**		
Based on equity	4.21	0.59
Facilitate peer support	4.29	0.62
**Collective domain**		
Facilitate collaborative learning environment	4.12	0.68
Based on bottom-up co-creation	3.88	0.80
**Organizational domain**		
Accessible to everyone	4.08	0.58
Position as equal partner	3.79	0.88
Contribute to destigmatization	3.75	0.79
Quality assurance	3.62	0.82
**Practical choices**		
Program scope	4.12	0.90
Co-creation partnerships	4.25	0.99
Location type	3.17	1.61

Core tasks were scored on a 5-point Likert scale. Practical choices were rated on a 5-point categorical scale

##### RCs Labeled as ‘RCs’ vs. Other Labels

Given the variation in labels used in the Dutch RC landscape, we explored whether initiatives that used the RC label (*N =* 12) differed in their tool scores as compared to peer-supported recovery initiatives that identified differently (*N =* 12). Shapiro-Wilk test illustrated that not all data was normally distributed. Out of the 20 tests (10 items, 2 groups), 16 were significant (p’s <.05, adjusted p-values with Benjamini and Hochberg (2000) correction to correct for multiple testing). Therefore, Mann-Whitney U-tests were conducted as non-parametric alternative of independent samples t-tests (Mann & Whitney, 1947; Wilcoxon, 1945; Table 4). On average, initiatives that adopted the RC label reported higher scores on ‘Quality assurance’, ‘Position as equal partner’, ‘Employ (methodological) self-help’, and ‘Contribute to destigmatization’, though these differences were not statistically significant (all adjusted p’s ≥ 0.27, adjusted with Benjamini and Hochberg (2000) correction). Effect sizes of these non-significant differences were moderate (*r* ranged from 0.25 to 0.46), indicative of potential practically significant differences among initiatives that do vs. do not adopt an RC label.

**Table 4:** Mann-Whitney U-tests comparing RC-labeled vs. other-labeled initiatives

	RC label (*N =* 12)		Other label (*N =* 12)		RC label vs. other label				
	*Mean*	*SD*	*Mean*	*SD*	*ΔMean*	*W*	*p*	*Adjusted p*	*Effect size (r)*
Self-direction	4.33	0.49	4.08	0.67	0.25	86	0.36	0.68	0.19
(Methodological) self-help	4.42	1.00	4.08	0.67	0.34	96	0.14	0.47	0.31
Equity	4.25	0.62	4.17	0.58	0.08	77.5	0.74	0.83	0.08
Peer support	4.25	0.62	4.33	0.65	− 0.08	66.5	0.74	0.83	0.07
Collaborative learning	4.00	0.74	4.25	0.62	− 0.25	58.5	0.41	0.68	0.18
Co-creation	3.83	0.94	3.92	0.67	− 0.09	70	0.93	0.93	0.03
Accessible	4.00	0.60	4.17	0.58	− 0.17	62	0.51	0.73	0.14
Equal partner	4.08	0.79	3.50	0.91	0.58	96.5	0.14	0.47	0.31
Destigmatization	3.92	0.79	3.58	0.79	0.34	90.5	0.24	0.61	0.25
Quality assurance	4.00	0.43	3.25	0.97	0.75	106.5	0.03	0.27	0.46

## Discussion

This study aimed to define core elements of Dutch RCs (RQ1), and how they differ from RCs as defined by the UK Fidelity Measure (RQ2). With the ambition to contextualize the Fidelity Measure to the Dutch context, another goal was to establish fidelity of Dutch RCs (RQ3), and to explore whether initiatives labeled as ‘RC’ conceptually differed from peer-supported recovery initiatives adopting other labels (RQ4). However, rather than translating a fidelity measure, the primary findings of this study led to the development and evaluation of a Reflection Tool for RCs instead. This reflection tool was valued for providing insights into the status quo and future development opportunities and received favorable ratings in terms of comprehensiveness, usability, acceptability, and content validity. The findings of this study contribute to describing Dutch RC practice (RQ1 and RQ4) and comparing this to the international RC model (RQ2).

### Dutch RC Practice: Varied with Common Ground

Our study found that, despite practical differences (e.g., co-creation partnerships, location types, and offerings), a common ground existed among Dutch RCs, as the core values and tasks of the reflection tool were widely recognized and aligned with practice. One core value among this was free space, representing a new concept in international RC literature. In Dutch conceptualizations and practice descriptions, free space is elaboratively defined as a multi-layered concept encompassing intrapersonal, interpersonal, emancipatory and organizational elements (Boertien & Harmsen, 2017; van Erp et al., 2023). Central is its liberating nature, which frees people from restrictions and oppression, creating space for their true selves. This allows individuals to explore relations with themselves and others, and to shape their recovery journey (and lives) aligned with their preferences. To facilitate this free space, organizational free space was considered crucial, such that the RC is co-created bottom-up without unwanted interference from external organizations or funders, like healthcare organizations or municipalities. Control over and ownership of activities, policies, and decisions within the RC should always belong to the individuals involved (Boertien & Harmsen, 2017). Alongside the emphasis on bottom-up co-creation, free space therefore contributed to the diversity of Dutch RC practices. The developed reflection tool accommodates this diversity of initiatives carrying various labels and emphasizing different core tasks in their practices. While in this article we decided to adopt the international acknowledged term ‘RC’, we suggest that, based on these findings, the reflection tool could also be relevant for a broader range of peer-supported recovery initiatives.

Though not statistically significant, some practically significant differences were observed when comparing initiatives that adopt the RC label with those that do not. Initiatives adopting the RC label scored higher on employing (methodological) self-help. Besides, labeled RCs seemed to be better equipped to engage in organization related core tasks, such as positioning as equal partners, contributing to destigmatization and participating in structural quality assurance (as reflected in their higher average scores). While an explanation for these differences is difficult to formulate, we can highlight observed differences between the two groups that may be relevant. We observed that RC-labeled initiatives were more frequently hosted by mental health services or sheltered living and supported housing organizations, while initiatives with different labels more often had client-driven origins (not reported in results section). Therefore, explaining any differences among initiatives adopting different labels requires further investigation.

### Are Dutch RCs Something Different?

Some deviations became apparent that may arguably result in a different RC practice in the Netherlands as compared to for example in the UK. The most outstanding difference regards the way co-creation is given shape, which is an essential element according to Toney et al. (2019). According to these authors, RCs in principle co-produce their offerings with mental healthcare providers, and therefore cannot be considered peer-led. This, however, was the most common format of Dutch RCs, where a peer-run philosophy was highly valued. Facilitating peer support was the core task that received the highest scores in the reflection tool. Moreover, descriptive information highlighted that Dutch RCs on average employed more volunteers than paid experts by experience or other employees, with some RCs even depending largely on voluntary contributions. Where RCs following the UK model attempt to catalyze culture change within mental healthcare services by collaborating with them, Dutch RCs seem to foster change by providing an alternative ‘space to be’ instead.

Another observed difference related to the focus on learning, which was emphasized both in the UK Fidelity Measure and the developed reflection tool, yet differing in nuance. ‘Employing (methodological) self-help’ was highly scored and can be considered comparable to the ‘Learning’ component of the UK Fidelity Measure, as methodological self-help tools such as the WRAP (Cook et al., 2011) are designed within an educational framework. A nuanced difference though lies within another component of the reflection tool about facilitating a collaborative learning environment, focusing on both formal and informal learning settings. Its high score reflected the wider scope of Dutch RCs beyond a curriculum (of methodological self-help) alone.

Despite these differences, Dutch RCs embraced recognizable elements that are described in international RC literature, such as a focus on equity, empowerment, exchange of experiences among peers, and facilitating a learning environment (Hayes et al., 2023b; Toney et al., 2019). This paper is not the first to reveal nuanced differences in RCs: Hayes et al. (2023a) for example distinguished strengths-oriented and community-oriented RCs. Aligned with this literature, we conclude that Dutch RCs are *not* something different, but rather exemplify the flexibility and diversity found in RCs internationally.

### Fidelity of a Flexible Practice: Theoretical and Practical Implications

This study not only corroborates the diversity of RCs, it also makes two main contributions, each with distinct implications: one theoretical, and one practical. Theoretically, the findings challenge the boundaries of fidelity in bottom-up, co-created settings such as RCs. RCs can be given shape in various ways, depending on for example healthcare context, funding possibilities and culture (Anfossi, 2017; Hayes et al., 2023b; King & Meddings, 2019). Even more so, free space to shape RC practice through co-creation lies at the very heart of the RC philosophy. Flexibility and diversity are thus inherent to RC practice, yet in the domain of fidelity ‘program drift’ cautions against deviations that dilute an intervention’s original model (Mowbray et al., 2003). This raises a key theoretical question: can we view RCs as a single model with varying ‘drifts’ (and if so, when are ‘drifts’ too strong?), or is the adaptable nature of RCs resistant to such definition? We raise this question in the context of recent developments in fidelity literature. Initially, fidelity was framed as an instrumental concept focused on the objectification of measurable constructs, but it has gradually expanded to accommodate qualitative interpretation too, allowing room for flexibility in operationalizations (e.g., in FACT: Westen et al., 2023, see also Teague et al., 2012). Analysis of empirical data in the RC context led us to develop a reflection tool which is not instrumental but facilitates a collaborative reflection process to enhance quality assurance. This perspective sheds new light on the developments in the fidelity domain.

Practically, the reflection tool enables RCs to engage with questions of quality in a way that aligns with their context-driven and co-developed character. The overview of core tasks can aid RCs to prioritize what aligns best with peers’ bottom-up needs in their specific context. Specifically, the reflective dialogues that the tool provokes can contribute to the safeguarding of an RC’s goals and values, for example in the light of external demands or (political) developments. Also of practical value is the tool’s co-creative application, fostering reflection in a way that aligns with RCs’ values of equity and bottom-up organization.

### Limitations

While this study offers valuable insights and contributes meaningfully to the field, it is important to acknowledge its limitations. One notable limitation is its relatively small sample size in the testing phase, due to a low response rate. The comprehensiveness of the reflection tool was appreciated for facilitating a profound exchange of what is deemed important and valuable; however, it also placed considerable demands on participants. It was repeatedly mentioned that a 1.5-hour session was short in time to successfully hear all voices and discuss all core tasks in-depth. Furthermore, RCs frequently expressed a desire to achieve more (when a core task received a lower score, for example) but were constrained by limited time and resources. It is imaginable that this strain also extended to insufficient time to participate in intensive research.

In addition to these practical barriers, the political context in the Netherlands at the time of this study may have also affected participation. The implementation of RCs and similar initiatives has been a topic of recent political attention and debate. This made the RC practice dynamic and developments unfolded rapidly. As a result, some participants were hesitant to engage in academic research aimed at describing the core of their practice, at this stage. It is possible that RCs whose ideology aligned with the scope of this study were more eager to participate, which could suggest a potential selection bias. However, the diversity in organizational characteristics among the participating RCs suggests that participation was not limited to one ‘type’ of RC. That said, conclusions on common grounds and comparisons between initiatives should still be interpreted with some caution.

### Future Directions

Building on the findings of this study, several avenues for future research can be identified. First, research should explore whether a reflection tool as developed in this study is of added value to the existent UK Fidelity Measure (Toney et al., 2019). Interesting questions would be whether reflecting on one’s practice differs when guided by a reflection tool versus a traditional fidelity measure, and how this affects further development of RCs. Testing and evaluating the tool in other contexts, such as other peer-supported recovery initiatives, may also help improve the tool’s accessibility in terms of language and format, to facilitate equal contributions of partakers from all levels even better. This allows exploration of the tool’s potential contribution to a diverse mental health support landscape.

Moreover, our study highlighted a conceptually different approach to co-production (or co-creation), raising questions about the comparability of RC research internationally (despite a common ground of core values). Future work should, therefore, investigate whether RCs with a different focus or different way of working have different impacts and outcomes, for example regarding (cost-)effectiveness. Such exploration may also focus on differences in RC experience on individual level, or organizational needs. Core tasks as specified in the reflection tool may aid such comparisons. Such investigation could further improve our understanding of how RCs impact those involved in various ways.

In any future research activity, co-creative and participatory methods, such as Participatory Action Research (PAR), should be incorporated to ensure that experiential knowledge is not only acknowledged but actively shapes the research process.

## Conclusion

In this study, a reflection tool for RCs was developed and evaluated. It underscored a common ground of values among RCs internationally, yet also highlighted conceptual differences in Dutch RCs as compared to RCs following the UK model. Beyond its empirical findings, the study’s key contribution lies in the critical reflection it provokes on the applicability of fidelity-based models within the context of RCs. By introducing and testing a reflective tool, the study highlights the potential of such instruments to serve as alternative or complementary quality measures that align with the adaptive, bottom-up nature of co-production in RCs.

## Supplementary Information

Below is the link to the electronic supplementary material.

ESM 1 (PDF 1.43 MB)

## Footnotes

1 Despite the variance of labels used in Dutch practice, for readability in this paper we refer to all initiatives that facilitate recovery-oriented self-help education, are accessible to all, and operate based on experiential knowledge, as RCs.

2 Although a ‘Dutch’ version is publicly available, this translation originates in Flanders (Belgium) and further revisions were necessary to better align Dutch language.

3 The original formulation of RQ2 referred to “the model of Dutch RCs.” However, given the flexible and co-created nature of RCs, we acknowledge that the term “model” may be misleading. This paper does not aim to define a model, but rather to explore how Dutch RCs relate to the UK fidelity measure in practice.

4 Co-production and co-creation are concepts that are frequently used interchangeably (while they may have different connotations). In international literature ‘co-production’ more frequently occurs, while in the Netherlands ‘co-creation’ is a more frequently used term. To align with Dutch practice, in this article we use the term ‘co-creation’ to refer to any process involving collaboration among individuals with (mostly) different roles.

5 Peers and mental healthcare providers are not mutually exclusive: peers can have educational backgrounds in mental healthcare, and mental healthcare providers can have lived experiences with mental health challenges.
