# Supplementary material for: Recovery Colleges or Something Different? The Development and Evaluation of a Reflection Tool for Recovery Colleges in the Netherlands
Source: Community Ment Health J. 2025 Oct 29;62(2):231–44. doi: 10.1007/s10597-025-01517-1 (PMC12852231; doi:10.1007/s10597-025-01517-1)

Appendix A – RECOLLECT Fidelity Measure (original and Dutch translation)

RECOLLECT Fidelity Measure for Recovery Colleges

Please note that the RECOLLECT Checklist is copyrighted and must not be changed without permission.
Further information: researchintorecovery.com/recollect

PART 1

INSTRUCTIONS
Part 1 produces a numerical score indicating the extent to which your Recovery College matches our best understanding of an ideal Recovery College. The score ranges from 0 (low fidelity) to 14 (high fidelity).<br><br>This Measure is completed by one or more people who have an overview of the Recovery College, e.g. Recovery College manager and/or peer trainer. Only complete this Measure for one Recovery College (even if involved in or managing more than one). By 'students' we mean all students using the college, not just those who use mental health services.<br><br>The following pages list seven key dimensions of a Recovery College. Each dimension has three statements describe varying levels of development, from early stage to active engagement to active success. For each dimension, **please TICK the statement which best matches your views and experience of your Recovery College.**

DIMENSION	CURRENT STAGE OF DEVELOPMENT	ANSWER *(tick)*
**1. Valuing equality**<br>The contributions and assets of students, trainers (peers, clinicians, external) and other staff are equally valued. No one is judged or treated differently because of their background or mental health difficulties.	We recognise that staff and students may take time to develop partnership-based working relationships. Whilst being supportive of staff and students, we only deal with issues of discrimination and power differences when they arise.	◯ 0
	We do not actively ensure that all relationships in the college demonstrate equal sharing of opportunities, training, etc. However, we do ensure that the college is welcoming to all staff and students, and have some structures in place (e.g. open days, training, supervision) to encourage equality and to challenge stigma and discrimination.	◯ 1
	We actively promote a non-judgemental and welcoming culture. Activities are undertaken to ensure that issues of power are always considered within the college (e.g. equal access to training and resources, diversity in promotional materials, analysing equal opportunity data).	◯ 2

DIMENSION	CURRENT STAGE OF DEVELOPMENT	ANSWER *(tick)*
**2. Learning**<br>Recovery Colleges follow an adult education approach whereby students and trainers collaborate and learn from each other by sharing experiences, knowledge and skills. Students have responsibility for their learning and learn through interactive and reflective exercises. Students gain self-awareness, understanding of their difficulties and practical, relevant self-management skills. Students choose courses which best suit their interests and aspirations.	We cannot provide evidence of the college's model(s) of adult learning. We can identify a large number of barriers to progress, such as the influence of a strong clinical or psychoeducational model, or limited resources for Peer Trainer training. Trainers are skilled in delivering education and encouraging shared learning.	◯ 0
	We can articulate the college's model(s) of adult learning. Some processes are in place to ensure that trainers follow educational principles (e.g. lesson plans, educational language) and that courses involve co-learning. However, some barriers prevent the full and effective implementation of these model(s), e.g. time pressures to launch/recruit to new courses, or barriers to trainer recruitment and training.	◯ 1
	We can demonstrate the college's full commitment to principles of adult learning. These are evident in the college's prospectus, curriculum and course materials. All trainers (including clinical trainers) can describe the model(s) of adult learning used in the college, and are offered ongoing formal or accredited training in adult learning.	◯ 2

DIMENSION	CURRENT STAGE OF DEVELOPMENT	ANSWER *(tick)*
**3. Tailored to the student** Recovery Colleges don't offer a onesize-fits-all experience. Students' individual needs are actively enquired about and accommodated during courses (e.g. personalised handouts, translated text, materials adapted for learning difficulties). Their needs outside the course are also accommodated (e.g. buddy service, transport help, individual learning plans).	We are not able to demonstrate the ways in which the college provides an individualised experience for students. Trainers are not actively supported or trained to take account of and accommodate student differences during classes.	◯ 0
	We can demonstrate some ways in which individual needs of students are addressed, but recognise that there are still unmet needs, e.g. students with learning difficulties or nonfluent English speakers.	◯ 1
	We are able to demonstrate many ways in which students' individual needs are addressed both during and outside courses. Trainers are made aware of students' needs in advance and provided with guidance on how to adapt the content/delivery of courses.	◯ 2

DIMENSION	CURRENT STAGE OF DEVELOPMENT	ANSWER *(tick)*
**4. Co-production of the Recovery College** People with lived experience (Peer Trainers and students) are brought together with professionals and subject experts to design and deliver all aspects of the Recovery College. This includes collaborative decisionmaking about the prospectus, courses, college policies, staff recruitment, advertising, etc., as well as the co-design and co-delivery of all courses by a Peer Trainer and other subject-expert.	We routinely involve students and staff in decision-making about the design and running of the Recovery College. Most of our success in co-production has been at the level of course co-delivery. We recognise that there are currently some significant barriers to co-production throughout the college, including those of culture, management hierarchies and time.	◯ 0
	As well as consistent co-delivery of courses, we involve staff and students in most discussions about the design and running of the Recovery College (e.g. through student steering groups or student reps), but managers make many of the decisions.	◯ 1
	We can demonstrate a culture of co-production and its consistent use across the college. The voices of trainers and students are equally heard during decision-making across all levels of the college, including co-delivery, curriculum development, management and design of the physical environment.	◯ 2

DIMENSION	CURRENT STAGE OF DEVELOPMENT	ANSWER *(tick)*
**5. Social connectedness**<br>Both the culture and the physical environment of the college provide students with opportunities to develop connections with others. The learning space is relaxed, e.g. nonclinical chair layout, access to drinks facilities, shared spaces for socialising. Trainers recognise and cater for students' social needs, e.g. organising exercises and breaks for chatting, sharing experiences and developing friendships.	Students' social experience is low on the Recovery College's agenda when deciding on course structure and the physical environment. There are no specific processes for students to get to know one another. Course venues rarely have facilities or spaces outside the classroom where students can relax or socialise.	◯ 0
	We ensure that the Recovery College is a welcoming environment for students. Trainers are encouraged to provide opportunities for socialising during courses where possible, but this is not central to their role. A few of our course spaces have facilities outside the classroom where students can relax, but there are a number of practical or financial barriers to this.	◯ 1
	The Recovery College recognises the role that student integration and connectedness plays in learning and recovery. The college provides a range of facilities for socialising (e.g. café, seating areas, informal and spacious course venues). Trainers are supported to integrate opportunities for students to form closer bonds with each other into the structure of courses.	◯ 2

DIMENSION	CURRENT STAGE OF DEVELOPMENT	ANSWER *(tick)*
**6. Community focus**<br>Recovery Colleges engage with community organisations (e.g. mental health charities, artistic/sporting groups) and Further Education colleges to co-produce relevant courses. The college provides students with information, handouts and events which support students' pathways into valued activities, roles, relationships and support in the community.	We have limited involvement with, or presence in, community organisations. Community organisations are not involved in college meetings or events, or do not routinely work with the college to co-produce courses or facilitate opportunities for staff/students.	◯ 0
	We ensure that the college undertakes some activities to build awareness of its community services and relationships with community organisations. Some college courses are coproduced with community organisations and students are signposted to relevant community organisations for support.	◯ 1
	We work with a range of community organisations to co-produce college courses and facilitate pathways for students. We can demonstrate activities to build awareness of, and relationships with, the community. We can demonstrate that joint-working with community organisations has led to changes in the college.	◯ 2

DIMENSION	CURRENT STAGE OF DEVELOPMENT	ANSWER *(tick)*
**7. Commitment to recovery**<br>Recovery College workers talk with conviction and enthusiasm about the service and are dedicated to students' recovery. There is a positive energy in the college and its activities, based on shared values about the recovery principles on which the college is based.	Our organisational policies and procedures ensure the Recovery College runs smoothly, but there are barriers (e.g. culture, organisational structures) to personal investment by workers in promoting recovery principles (dimensions 1 to 6 above) throughout the college. There is still significant effort needed to establish the college as something 'different' and 'meaningful'.	◯ 0
	We actively motivate each other to promote recovery principles. We have a shared commitment to constantly improve the recovery focus of the college but recognise some barriers to progress (e.g. cultural, financial).	◯ 1
	We actively promote recovery principles in the college, and collectively lead with enthusiasm and an expressed belief in the college's students and staff. College activities demonstrate recovery principles in practice, e.g. graduation ceremonies, students becoming trainers.	◯ 2

PART 2

How to complete this section
Part 2 characterises five further components of Recovery Colleges. For these components, it is not known which of the two types is better, so there is no best answer. We recognise that Recovery Colleges are complex and often span both types, so please pick the type that most closely resembles your college.<br><br>For each component below, **please identify whether your college is more like TYPE 1 or TYPE 2 by ticking ONE statement for each component.**

COMPONENT	TYPE	ANSWER *(Tick TYPE I or TYPE II)*
**8. Available to all**<br>Recovery Colleges vary in the ways in which they implement eligibility criteria for student access.	**The Recovery College is available to all.**<br><br>The Recovery College is accessible to any adult (16+ or 18+), including staff and carers, regardless of their use of local services of any kind. Any restrictions are minimal, e.g. living locally, being registered with a GP.	◯ TYPE I

	**The Recovery College is limited to specific groups.**<br><br>The Recovery College is open to adults (16+ or 18+) who are current or previous users of local secondary care mental health services. There may be local additions to this eligibility e.g. health/social care/community organisation staff, or family and carers. Being 'inclusive' relates to the ways in which the Recovery College does not discriminate or create access barriers for people with, for example, certain diagnoses, learning difficulties or physical health/mobility needs.	◯ TYPE II

COMPONENT	TYPE	**ANSWER** *(Tick TYPE I or TYPE II)*
**9. Location**<br>Recovery Colleges vary in where courses are run.	**The Recovery College is based in a community location that is not shared with health, social care or other statutory services.**<br><br>The Recovery College is deliberately located within communities or neighbourhoods, not in NHS or social care buildings.	◯ TYPE I
	**The Recovery College is based in a location which is shared with health, social care or other statutory services.**<br><br>The Recovery College is located within or near (e.g. adjoining building) to local NHS or social care services.	◯ TYPE II

COMPONENT	TYPE	**ANSWER** *(Tick TYPE I or TYPE II)*
**10. Distinctiveness of course content**<br>Recovery Colleges vary in the content/subjectmatter of courses offered.	**Any topic can be offered as a course, irrespective of whether it is available in mainstream adult education settings.**<br><br>The curriculum includes courses on topics which are also available in local mainstream colleges. Example courses might include gardening, arts, Maths, English, budgeting, understanding benefits, physical health care, job-seeking, home maintenance and a range of leisure/recreation activities.	◯ TYPE I

	**Only topics not available in mainstream adult education settings are offered.**<br><br>The curriculum never includes courses on topics which are available in local mainstream colleges. Some courses are offered with a specific recovery-related focus, e.g. gardening for wellbeing, arts for recovery.	◯ TYPE II

COMPONENT	TYPE	ANSWER *(Tick TYPE I or TYPE II)*
**11. Strengths-based**<br>A strengths-based approach (focussing on assets and potential, not on problems) is either explicit or implicit within the language, courses and materials of the Recovery College.	**A focus on strengths (not problems) is implicit in the college.**<br><br>The learning opportunities offered by the Recovery College implicitly builds on the experiences, strengths, assets and resources of students. The language of being 'strengths-based' is not often used.	◯ TYPE I
	**A focus on strengths (not problems) is explicit in the college, in addition to dimensions 1-7 above.**<br><br>The learning opportunities offered by the Recovery College explicitly build on the experiences, strengths, assets and resources of students. The language of being 'strengths-based' is routinely used by staff and students, and features in course materials and other aspects of the Recovery College.	◯ TYPE II

COMPONENT	TYPE	ANSWER *(Tick TYPE I or TYPE II)*
**12. Progressive**<br>There is variation in the ways in which Recovery Colleges focus on, enable and encourage the forward-moving, goal-focused nature of the student experience.	**There is a focus on 'being' and 'belonging', not on goal-setting.**<br><br>The focus of the Recovery College is on supporting individual students' learning needs, safety and belonging, identity development, personal meaning-making and reflection. The college does not require behavioural goal-setting. Students can learn in whatever direction they want to – and for some students that might not be about moving forwards.	◯ TYPE I

	**There is a focus on 'becoming' and a strong emphasis on goal-setting and change.**<br><br>The focus of the Recovery College is on processes which provide pathways of opportunity for students and which support them to move on with their lives. This might include the use of goal-oriented personal plans (Individual Learning Plans) and planning and reviewing goal-oriented activities.	◯ **TYPE II**

RECOLLECT Fidelity Measure (Dutch)

Nederlandse Modelgetrouwheidsschaal voor Herstelacademies

Let op: de RECOLLECT checklist is auteursrechtelijk beschermd en mag niet aangepast worden zonder toestemming.
Voor verdere informatie: researchintorecovery.com/recollect

DEEL I

INSTRUCTIES
In Deel 1 wordt een numerieke score samen gesteld die de mate weergeeft waarin jouw herstelacademie aansluit bij ons begrip van een ideale herstelacademie. De score loopt van 0 (lage modelgetrouwheid) tot 14 (hoge modelgetrouwheid).<br><br>Deze vragenlijst wordt ingevuld door een of meer personen die een overzicht hebben van de herstelacademie, bv. de coördinator van de herstelacademie en/of ervaringsdeskundige trainer. Vul deze vragenlijst alleen in voor één herstelacademie (ook al ben je betrokken bij of coördineer je verschillende herstelacademies). Met 'studenten' bedoelen we alle mensen die gebruik maken van de academie, niet alleen degenen die gebruik maken van geestelijke gezondheidszorg.<br><br>Op de volgende pagina's staan zeven belangrijke kenmerken van een herstelacademie. **Bij elk kenmerk staan drie antwoordopties. Hoe hoger de score, hoe meer het kenmerk terugkomt in de herstelacademie. Kruis voor elk kenmerk het antwoord aan dat het beste overeenkomt met jouw herstelacademie.**

KENMERK	ONTWIKKELINGSSTADIUM	**ANTWOORD** *(Kruis aan)*
**1. Er is aandacht voor gelijkwaardigheid**<br>Studenten, trainers (peers, ggz-professionals,<br>externen) en andere medewerkers kunnen op een gelijkwaardige manier bijdragen aan de herstelacademie. Er is ruimte voor de krachten en talenten van iedereen. Niemand wordt anders beoordeeld of behandeld vanwege hun<br>achtergrond of psychische kwetsbaarheid. Dit betekent ook dat we een open cultuur nastreven waarin iedereen zich welkom voelt.	Wij begrijpen dat medewerkers en studenten tijd nodig hebben om gelijkwaardige werkrelaties op te bouwen. We bieden hierbij ondersteuning aan zowel studenten als medewerkers, maar we reageren alleen op gevallen van discriminatie en machtsongelijkheid wanneer deze zich voordoen.	◯ 0
	Wij zorgen er niet actief voor dat alle relaties binnen de herstelacademie gelijkwaardig zijn (bijvoorbeeld op het gebied van kansen, training, etc.). Wij zorgen er echter wel voor dat alle medewerkers en studenten zich welkom voelen in de herstelacademie. En dat er een structuur aanwezig is (bv. open dagen, opleidingen, supervisie) om gelijkwaardigheid aan te moedigen. Zo gaan we ook stigmatisering en discriminatie tegen.	◯ 1
	Wij zetten ons actief in voor een oordeelvrije en gastvrije cultuur. Er worden activiteiten ondernomen om ervoor te zorgen dat er altijd aandacht is voor machtskwesties binnen de herstelacademie (bv. gelijke toegang tot opleiding en ondersteuning, diversiteit in promotiemateriaal, het analyseren van gegevens omtrent gelijke kansen).	◯ 2

KENMERK	ONTWIKKELINGSSTADIUM	**ANTWOORD** *(Kruis aan)*
**2. Leren centraal**<br>De herstelacademie werkt volgens de aanpak van het volwassenenonderwijs. Studenten en trainers werken samen en leren van elkaar door het delen van ervaringen, kennis en vaardigheden. Studenten zijn verantwoordelijk voor hun eigen leerproces. Ze leren door samen te oefenen en worden aangezet	We kunnen niet laten zien dat de academie werkt volgens de aanpak van het volwassenenonderwijs. Wij kunnen hiervoor veel hindernissen aanwijzen. Zoals de invloed van een klinische aanpak, of een psycho-educatieve aanpak (gericht op leren over je kwetsbaarheden als onderdeel van behandeling). Beperkte ondersteuning voor de opleiding van Peer Trainers is ook een hindernis. Trainers zijn wel toegerust om onderwijs te geven en samen leren aan te moedigen.	◯ 0
	Wij kunnen laten zien dat de academie werkt volgens de aanpak van het volwassenenonderwijs. Er zijn enkele processen aanwezig om ervoor te zorgen dat trainers de onderwijsprincipes volgen (bv. lesplanning, gebruiken van educatief taalgebruik). En dat in de cursussen gebruik gemaakt wordt van samen leren. Toch zijn er een aantal hindernissen die volledig werken volgens de aanpak van het	◯ 1

tot nadenken. Studenten ontwikkelen zelfbewustzijn. Ze krijgen inzicht in hun eigen moeilijkheden. En ze ontwikkelen vaardigheden voor zelf management. Studenten kiezen cursussen die het best passen bij hun interesses en doelen.	volwassenenonderwijs tegenhouden. Zoals tijdsdruk om nieuwe cursussen op te starten, of problemen om nieuwe trainers te vinden en op te leiden.	
	Wij kunnen laten zien dat de academie volledig werkt volgens de aanpak van het volwassenenonderwijs. Dit komt duidelijk naar voren in de doelstellingen, het programma-aanbod en het cursusmateriaal van de academie. Alle trainers (inclusief klinische trainers) kunnen de principes van volwassenenonderwijs omschrijven die gebruikt worden in de academie. Ze krijgen doorlopende erkende opleidingen rond volwassenenonderwijs aangeboden.	◯ 2

**KENMERK**	**ONTWIKKELINGSSTADIUM**	**ANTWOORD** *(Kruis aan)*
**3. Aanbod is op maat van de student** Herstelacademies bieden geen one-size-fits-all ervaring. Er wordt actief gevraagd naar de persoonlijke behoeften van studenten. En daar wordt rekening mee gehouden tijdens de cursussen (bv. aangepaste hand-outs, vertaling van teksten, materiaal aangepast aan leerproblemen). Ook met hun behoeften buiten de cursussen wordt rekening gehouden (bv. maatjes-dienst, hulp bij vervoer, individuele leerplannen).	Wij kunnen niet laten zien op welke manieren de academie aan studenten een persoonlijke ervaring biedt. Trainers worden niet actief ondersteund of opgeleid om met de verschillen tussen studenten rekening te houden. En om daarmee om te gaan tijdens de cursussen.	◯ 0
	Wij kunnen een aantal manieren laten zien waarop we rekening houden met persoonlijke behoeften van studenten. Maar we erkennen dat er ook nog steeds behoeften zijn waarmee geen rekening wordt gehouden. Bv. studenten met leerproblemen of studenten die de Nederlandse taal niet vloeiend spreken.	◯ 1
	Wij kunnen veel voorbeelden geven van manieren waarop we rekening houden met de persoonlijke behoeften van studenten, zowel tijdens als buiten de cursussen. Trainers worden vooraf verteld wat de behoeften van studenten zijn. En trainers krijgen begeleiding bij het aanpassen van de inhoud en de uitvoering van de cursussen.	◯ 2

**KENMERK**	**ONTWIKKELINGSSTADIUM**	**ANTWOORD** *(Kruis aan)*
**4. Co-creatie van de herstelacademie** Mensen met ervaringskennis (zowel peer trainers als studenten) geven de herstelacademie vorm,	We betrekken studenten en medewerkers regelmatig bij het nemen van beslissingen over hoe de herstelacademie eruit ziet. We zijn vooral succesvol in co-creatie als het gaat over het samen faciliteren van de cursussen. We zijn ons er van bewust dat er momenteel nog een aantal grote hindernissen zijn voor co-creatie in alle onderdelen van de academie. Zoals de cultuur, hiërarchie in het bestuur van de academie en gebrek aan tijd.	◯ 0

samen met ggz-professionals en mensen met een specifieke expertise. Dit gebeurt in alle onderdelen van de academie. Er worden samen beslissingen genomen over het programma-aanbod, de cursussen, het beleid, het in dienst nemen van personeel, het maken van reclame, etc. Het houdt ook in dat alle cursussen samen worden ontwikkeld en gefaciliteerd door een trainer met ervaringskennis en een andere deskundige.	Naast het standaard samen aanbieden van cursussen, betrekken we medewerkers en studenten bij de meeste discussies over hoe de herstelacademie eruit ziet (bijv. via stuurgroepen voor studenten of studentenvertegenwoordigers). Maar coördinatoren nemen de meeste beslissingen.	◯ 1
	Wij kunnen laten zien dat we een cultuur van co-creatie hebben en hier ook standaard gebruik van maken in de hele herstelacademie. De stemmen van trainers en studenten worden op een gelijkwaardige manier gehoord tijdens het nemen van beslissingen. Dit gebeurt in alle onderdelen van de academie, zoals het co-faciliteren en het ontwikkelen van het programma-aanbod. En hoe de fysieke ruimte wordt vormgegeven en onderhouden.	◯ 2

**KENMERK**	**ONTWIKKELINGSSTADIUM**	**ANTWOORD** *(Kruis aan)*
**5. Aandacht voor sociale verbondenheid** De herstelacademie biedt studenten mogelijkheden om verbindingen aan te gaan met anderen. Dit komt door de cultuur en de fysieke omgeving van de academie. De leeromgeving is ontspannen. Bijvoorbeeld door een niet-klinische opstelling van stoelen, de mogelijkheid om een drankje te drinken, en de aanwezigheid van ruimtes waar je elkaar kunt ontmoeten. Trainers kennen de sociale behoeften van	De sociale ervaringen van studenten staan laag op de agenda van de herstelacademie bij het bepalen van cursusstructuur en de fysieke omgeving. Er zijn geen speciale mogelijkheden voor studenten om elkaar beter te leren kennen. Cursuslocaties hebben zelden faciliteiten of ruimtes buiten het klaslokaal waar studenten kunnen ontspannen of elkaar kunnen ontmoeten.	◯ 0
	Wij zorgen ervoor dat de herstelacademie een gastvrije omgeving is voor studenten. Trainers worden aangemoedigd om mogelijkheden te bieden voor sociaal contact tijdens de cursussen als het kan, maar dit is niet hun centrale functie. Een aantal van onze cursuslocaties hebben faciliteiten buiten het klaslokaal waar studenten zich kunnen ontspannen. Maar er zijn een aantal praktische of financiële belemmeringen hiervoor.	◯ 1
	De herstelacademie weet dat samenkomen en verbinding tussen studenten een rol speelt in leer- en herstelprocessen. De academie biedt veel verschillende faciliteiten aan gericht op ontmoeting (bv. café, zithoeken, gezellige en ruime cursuslocaties). Trainers worden aangemoedigd om mogelijkheden te bieden voor studenten om hechte banden met elkaar aan te gaan tijdens de cursussen.	◯ 2

studenten en spelen hier op in. Zij maken bijvoorbeeld gebruik van oefeningen en pauzes waarin men met elkaar kan praten. Zo kan men ervaringen delen en kunnen vriendschappen ontwikkelen.		

**KENMERK**	**ONTWIKKELINGSSTADIUM**	**ANTWOORD** *(Kruis aan)*
**6. Focus op de wijk**<br>Herstelacademies werken samen met organisaties uit de wijk om samen interessante cursussen te ontwikkelen. Denk bijvoorbeeld aan liefdadigheidsorganisaties, creatieve verenigingen, sportverenigingen en wijkcentra. De academie biedt studenten informatie, folders en evenementen aan die de studenten helpen hun weg te vinden naar betekenisvolle activiteiten, rollen, relaties en ondersteuning in de wijk.	Wij zijn slechts een beetje betrokken bij wijkorganisaties. We zijn er ook weinig aanwezig. Organisaties uit de wijk zijn niet betrokken bij bijeenkomsten of evenementen van de academie. Wijkorganisaties werken ook niet regelmatig samen met de academie om cursussen te ontwikkelen of kansen voor medewerkers/studenten te faciliteren.	◯ 0
	Wij zorgen ervoor dat het duidelijk is voor de wijk wat de academie doet en hoe ze samenwerkt met wijkorganisaties. Sommige cursussen worden in samenwerking met wijkorganisaties vormgegeven. Studenten worden ook doorverwezen naar nuttige wijkorganisaties voor ondersteuning.	◯ 1
	We werken samen met verschillende wijkorganisaties om cursussen en leerpaden voor studenten vorm te geven. We kunnen laten zien dat we dingen doen om onze rol en samenwerking met de wijk op te bouwen. We zorgen dat het duidelijk is voor de wijk wat we doen en voor wie we zijn. We kunnen ook laten zien dat samenwerking met de wijkorganisaties heeft geleid tot veranderingen in de academie.	◯ 2

**KENMERK**	**ONTWIKKELINGSSTADIUM**	**ANTWOORD** *(Kruis aan)*
**7. Inzetten op herstel**<br>De medewerkers van de herstelacademie praten met overtuiging en enthousiasme over de organisatie. Zij zetten zich in	Ons organisatorisch beleid en onze procedures zorgen ervoor dat de herstelacademie goed functioneert. Er zijn wel een aantal belemmeringen die ervoor zorgen dat medewerkers minder actief betrokken zijn om de herstelprincipes te promoten (kenmerken 1 tot en met 6 hierboven) in de academie. Voorbeelden van die belemmeringen zijn de cultuur en de organisatiestructuur. Er zijn nog veel inspanningen nodig om de herstelacademie op de kaart te zetten als "anders" en “betekenisvol”.	◯ 0

voor het herstel van studenten. Er heerst een positieve energie rond de herstelacademie en de activiteiten. Die energie is gebaseerd op gedeelde waarden over de herstelprincipes die in de herstelacademie centraal staan.	Wij motiveren elkaar actief om herstelprincipes te promoten. We hebben een gedeelde inzet om de focus op herstel van onze academie steeds te verbeteren. Maar we zijn ons ook bewust van een aantal belemmeringen die er nog zijn (bv. cultureel, financieel).	◯ 1
	Wij promoten de herstelprincipes actief in de herstelacademie. We leiden gezamenlijk met enthousiasme en een uitgesproken geloof in de studenten en de medewerkers van de academie. De activiteiten van de academie laten de herstelprincipes zien in de praktijk. Bijvoorbeeld ceremonies bij afstuderen, studenten die trainers worden.	◯ 2

RECOLLECT Fidelity Measure (Dutch)

Nederlandse Modelgetrouwheidsschaal voor Herstelacademies

Let op: de RECOLLECT checklist is auteursrechtelijk beschermd en mag niet aangepast worden zonder toestemming.
Voor verdere informatie: researchintorecovery.com/recollect

DEEL 2

**Hoe je dit deel invult**
In Deel 2 worden nog vijf andere kenmerken van herstelacademies beschreven. Voor deze kenmerken is niet bekend welke van de twee types beter is, dus er is geen juist antwoord. We zijn ons ervan bewust dat herstelacademies complex zijn en vaak beide types omvatten, dus kies het type dat het *beste overeenkomt* met jouw herstelacademie.<br><br>**Geef voor elk kenmerk hieronder aan of jouw herstelacademie meer lijkt op TYPE 1 of TYPE 2 door voor elk kenmerk ÉÉN antwoord aan te kruisen.**

**KENMERK**	**TYPE**	**ANTWOORD** *(kruis TYPE I of TYPE II aan)*
**8. Beschikbaar voor iedereen**<br>Herstelacademies verschillen in de manier waarop ze toegangseisen gebruiken voor studenten.	**De herstelacademie is beschikbaar voor iedereen.**<br>De herstelacademie is beschikbaar voor alle volwassenen (16+ of 18+), inclusief begeleiding- en zorgmedewerkers. Het maakt niet uit of zij gebruik maken van lokaal ggz-behandelaanbod en begeleidingsaanbod. Het kan zijn dat er minimale eisen zijn, bv. dat je in de buurt moet wonen, of geregistreerd moet staan bij een lokale (huis)arts.	◯ TYPE I
	**De herstelacademie is alleen beschikbaar voor specifieke groepen.**<br>De herstelacademie is beschikbaar voor volwassenen (16+ of 18+) die huidige of vroegere cliënten zijn van lokale gespecialiseerde ggz-diensten. Dit kan aangevuld worden door bijvoorbeeld praktijkwerkers uit de ggz of sociale sector. Of familie en verzorgers. ‘Inclusief zijn’ gaat over de manieren waarop de herstelacademie niet discrimineert of hindernissen creëert voor mensen met bijvoorbeeld bepaalde diagnoses, leerproblemen of fysieke gezondheids- of mobiliteitsbehoeften.	◯ TYPE II

**KENMERK**	**TYPE**	**ANTWOORD** *(kruis TYPE I of TYPE II aan)*
**9. Locatie**<br>Herstelacademies verschillen in de plaats waar de cursussen worden gegeven.	**De herstelacademie maakt gebruik van een wijklocatie die niet gedeeld wordt met de gezondheidszorg, sociale diensten of andere overheidsdiensten.**<br>De herstelacademie is bewust onderdeel van lokale buurten en wijken. En maakt geen gebruik van gebouwen van de gezondheidszorg of sociale diensten.	◯ **TYPE I**
	**De herstelacademie maakt gebruik van een locatie die gedeeld wordt met de gezondheidszorg, sociale diensten of andere overheidsdiensten.**<br>De herstelacademie is onderdeel van een gebouw van de lokale gezondheidszorg of sociale diensten. Dit kan in eenzelfde gebouw zijn of in een aangrenzend gebouw.	◯ **TYPE II**

**KENMERK**	**TYPE**	**ANTWOORD** *(kruis TYPE I of TYPE II aan)*
**10. Mate waarin de cursusinhoud uniek is**<br>Herstelacademies verschillen in de inhoud/onderwerpen van het aanbod.	**Elk onderwerp kan als cursus worden aangeboden. Het maakt niet uit of dezelfde cursussen ook in het standaard cursusaanbod van wijkorganisaties beschikbaar zijn.**<br>In het programma-aanbod staan cursussen over thema's die ook beschikbaar zijn in de lokale wijkorganisaties. Voorbeelden van cursussen kunnen gaan over tuinieren, kunst, wiskunde, Nederlands, budgetbeheer, inzicht krijgen in uitkeringen, lichamelijke gezondheidszorg, het zoeken van werk, het huishouden en een reeks vrijetijdsactiviteiten.	◯ **TYPE I**
	**Er worden alleen onderwerpen aangeboden die niet beschikbaar zijn in het standaard cursusaanbod van wijkorganisaties.**<br>In het programma-aanbod staan nooit cursussen over thema's die beschikbaar zijn in de lokale wijkorganisaties. Sommige cursussen kunnen wel aangeboden worden met een specifieke focus op herstel, bijvoorbeeld tuinieren voor welzijn, kunst voor herstel.	◯ **TYPE II**

**KENMERK**	**TYPE**	**ANTWOORD** *(kruis TYPE I of TYPE II aan)*
**11. Focus op krachten**<br>Een krachtgerichte benadering (met een focus op krachten en potentieel, niet op problemen) is	**Een krachtgerichte focus (i.p.v. een focus op problemen) is impliciet aanwezig in de academie.**<br>De leermogelijkheden die de herstelacademie biedt zijn impliciet gericht op het versterken van de ervaringen, sterktes, krachten en ondersteuningsbronnen van de studenten. 'Krachtgericht' taalgebruik wordt niet vaak gebruikt.	◯ **TYPE I**

aanwezig in de herstelacademie. Die benadering is zichtbaar in het taalgebruik, de cursussen en de materialen van de herstelacademie. Deze krachtgerichte focus kan impliciet of expliciet aanwezig zijn.	**Een krachtgerichte focus (i.p.v. een focus op problemen) is expliciet aanwezig in de academie, naast kenmerken 1-7 die hierboven zijn opgesomd.**<br>De leermogelijkheden die de herstelacademie biedt zijn expliciet gericht op het versterken van ervaringen, sterktes, krachten en ondersteuningsbronnen van de studenten. De medewerkers en de studenten gebruiken regelmatig ‘krachtgerichte’ taal. Die ‘krachtgerichte’ taal komt ook terug in het cursusmateriaal en andere onderdelen van de herstelacademie.	◯ **TYPE II**

**KENMERK**	**TYPE**	**ANTWOORD** *(kruis TYPE I of TYPE II aan)*
**12. Doelgerichtheid**<br>Herstelacademies verschillen in de manier waarop ze focussen op de toekomst en het stellen van doelen. Het gaat hierbij over het benadrukken, faciliteren en aanmoedigen van een doelgerichte focus voor de studenten van de herstelacademie.	**De focus ligt op 'zijn' en 'erbij horen', niet op het stellen van doelen.**<br>De focus van de herstelacademie ligt op:<br>• het ondersteunen van de individuele leerbehoeften van studenten;<br>• veiligheid en erbij horen;<br>• ontwikkelen van je identiteit;<br>• persoonlijke betekenisgeving;<br>• nadenken (over jezelf).<br>De academie eist niet dat men persoonlijke doelen stelt. Studenten kunnen leren en groeien in de richting die ze zelf willen. Voor sommige studenten gaat dat misschien niet over vooruitgaan.	◯ **TYPE I**
	**De focus ligt op 'worden'. Er ligt sterke nadruk op doelgerichtheid en verandering.**<br>De focus van de herstelacademie ligt op het creëren van kansen die studenten helpen om verder te gaan met hun leven. Dit kan door doelgerichte persoonlijke plannen (individuele leerplannen) te gebruiken. Of door doelgerichte activiteiten te plannen en beoordelen.	◯ **TYPE II**

The RECOLLECT Checklists and Fidelity Measure were developed as part of the RECOLLECT Programme at University of Nottingham. Further information from researchintorecovery.com/recollect. This translation was published in 2025 by Marloes van Wezel (mwezel@trimbos.nl).

De RECOLLECT Checklists en Fidelity Measure zijn ontwikkeld als onderdeel van het RECOLLECT-programma aan de Universiteit van Nottingham. Meer informatie is te vinden op researchintorecovery.com/recollect. Deze vertaling werd gepubliceerd in 2025 door Marloes van Wezel (mwezel@trimbos.nl).

Distributed using Creative Commons License: CC BY-NC-ND 4.0.

Appendix B

Overview of initial themes and their relation to the UK fidelity measure

**Theme**	**Relation to UK fidelity measure (sub-codes)**
Strive for equity	• Inspired by '1. Valuing equality'<br>• No discrimination and welcoming culture relocated to 'Accessible for everyone'<br>• Add: Role fluidity<br>• Add: Reciprocity
Collaborative <u>learning environment</u> (peer-supported learning communities)	• Inspired by '2. Learning'<br>• Add: Focus on learning community<br>• Remove: References to adult education
<u>Collaborative</u> learning environment	• Inspired by '10. Distinctiveness of course content': peer support = distinctive<br>• Inspired by '5. Social connectedness'<br>• Add: Focus on peer supported community<br>• Add: Reciprocity
Bottom-up co-creation	• Inspired by '3. Tailored to the student'<br>• Inspired by '4. Co-production of the RC'<br>• Add: By and for peers<br>• Note: Relates to equity
Personal free space	• Related to '12. Progressive': both space for being and belonging, and becoming
Focus on self-direction and recovery	• Inspired by '7. Commitment to recovery'<br>• Inspired by '11. Strength based'
Accessible for everyone	• Inspired by '8. Available to all'<br>• Remove: Distinction between target groups<br>• Add: Low thresholds = key
Focus on the community/destigmatization/ emancipation?	• Inspired by '6. Community focus'<br>• Add: Focus on integration within society/neighborhood
Language use throughout tool	• Participants (instead of students)<br>• Facilitators (instead of trainers)<br>• Meeting, work group or series (instead of training/course)<br>• Student is not the end user (~bottom-up instead of top-down)

Appendix C

Reflection Tool for Recovery Colleges and Other Peer-Supported Recovery Initiatives

© April 2024, Tranzo, Tilburg University and the Trimbos-institute

Contents

1. Introduction ... 3
1.1. Background ... 3
1.2. How to use this tool ... 3
1.3. Role definition ... 4
2. Core values ... 5
Free space ... 5
Recovery ... 5
Empowerment ... 5
Experiential knowledge and expertise by experience ... 6
Peer support ... 6
Anti-stigma ... 6
User emancipatory movement ... 6
3. Core tasks – scoring instruction ... 7
3.1. Personal domain ... 7
Offer opportunities for self-direction ... 7
Employ (methodological) self-help ... 8
3.2. Relational domain ... 9
Based on equity ... 9
Facilitate peer support ... 9
3.3. Collective domain ... 10
Facilitate a collaborative learning environment ... 10
Based on bottom-up co-creation ... 10
3.4. Organizational domain ... 11
Accessible to everyone ... 11
Position as equal partner ... 12
Contribute to destigmatization ... 12
Quality assurance ... 13
4. Practical choices ... 14
Program offerings ... 14
Program scope ... 14
Co-creation partnerships ... 14
Type of location ... 15
5. Bibliography ... 17
6. Acknowledgments ... 18
7. Attachment – template radar tool ... 19

© April 2024, Tranzo, Tilburg University and the Trimbos-institute

1. Introduction

This is a reflection tool for Recovery Colleges and other peer-supported recovery initiatives, developed in the Netherlands (Dutch version available upon request). The tool describes important core values and core tasks. In the field of peer-supported recovery initiatives, various names are currently used (e.g., recovery college, recovery initiative, self-management center, etc.). This tool ignores those different names and focuses on the practice of initiatives that engage in **collaborative learning**, **methodical self-help**, **recovery**, and **peer support**.

The outcome of completing this tool is twofold:

1. The tool can be used for reflection, for example, for the further development of the initiative. It provides insight into how well important core values/core tasks are already woven into practice, and where there are still opportunities for development.
2. The tool describes what important characteristics are of Recovery Colleges in the Netherlands, making adequate research into these initiatives possible.

1.1. Background

This reflection tool has been developed as part of a doctoral research project on Recovery Colleges. The concept of 'Recovery Colleges' originally comes from Anglo-Saxon countries (United Kingdom, United States). The starting point for the development of this tool was an English Recovery College Fidelity Measure (Toney et al., 2019). However, the English measure required a significant translation before it aligned well with Dutch practice. The Dutch practice is more broadly oriented, and the English model centers on co-production with healthcare providers, which is not the case in the Netherlands. Although the starting point of this tool originally stemmed from the concept of the 'Recovery College', it has ultimately become more broadly applicable for peer-supported recovery initiatives in practice. Nonetheless, in the continuation of this tool, we refer to 'Recovery Colleges'.

To develop this reflection tool, focus groups were held with 30 peer facilitators and coordinators from 16 Recovery Colleges in the Netherlands. Existing descriptions and characteristic lists of peer-supported recovery initiatives were also used. Additionally, the experiential researchers from the POP Group contributed. 'POP' in 'POP Group' stands for Peer Researchers Perspective (*Peer Onderzoekers Perspectief* in Dutch). The POP group is a collective of participants, volunteers, and employees from Enik Recovery College who are involved in the doctoral research project. Lastly, experts from MIND, the Dutch Association for Self-Direction and Recovery, and Cavallo Advice (part of the 'Blauwe Paard Netwerk') also provided input.

Besides this tool, there are other lists describing the characteristics of peer-supported recovery initiatives, such as the list from the Workgroup accessible support centers (2023). That list shows many similarities with this tool. What distinguishes this present tool from other existing lists is that it identifies core values and core tasks of a Recovery College. These core values and core tasks are not only extensively described but also made measurable where possible.

1.2. How to use this tool

The tool is structured into three chapters. In the first chapter, a number of core values that are the basis of Recovery Colleges are described.

© April 2024, Tranzo, Tilburg University and the Trimbos-institute

In the second chapter, ten core tasks of Recovery Colleges are described. Scores have been assigned based on the degree to which a core task is reflected in the activities, policies, and decisions within a Recovery College. These scores are as follows:

1. Does not apply at all
2. Somewhat applies
3. Moderately applies
4. Largely applies
5. Applies completely

The tool is based on self-scoring by a coordinator or experienced peer trainer of a Recovery College. The results are made visible in two ways:

1. A visual overview in the form of a radar tool, which clearly shows at a glance the areas with significant emphasis and those with room for development (see attachment).
2. An online dashboard where bar charts are available for each level (personal, relational, collective, and organizational) that represent the status of the Recovery College. The higher the score on a core task, the better it (and the underlying core values) is integrated into practice.

To further address the identified areas for development in practice, the descriptions of the core tasks in this document can be helpful.

The third chapter highlights a number of practical choices. Sometimes there are various choices to make when shaping a Recovery College. One choice is not necessarily better or worse than another. Making choices visible and placing them alongside other possibilities can lead to new insights or ideas about shaping one's own Recovery College.

Although this tool is based on self-scoring, it is advisable to engage in the reflection process with others. Various forms can be considered for this. For the current research purposes, we envision the following two forms, with the first being our strong preference.

1. The reflection tool is filled out jointly by the coordinator(s), staff, volunteers, and participants in a meeting. Reflection occurs in dialogue, and the score is collaboratively determined.
2. The reflection tool is filled out by the coordinator(s), and the results are discussed with staff/volunteers/participants.

In this way, people can learn from each other, and more depth can be added to the reflection process.

1.3. Role definition

In this tool, reference is sometimes made to the partakers of the Recovery College as a whole, and sometimes to specific roles:

- Those involved (or partakers): Everyone who is actively involved in the Recovery College, regardless of their role.
- Participants: People who take part in activities of the Recovery College.
- Facilitators: People who facilitate activities within the Recovery College.
- Staff: People employed by the Recovery College.
- Volunteers: People who do volunteer work within the Recovery College.
- Visitors: People who visit the meeting space of the Recovery College.

© April 2024, Tranzo, Tilburg University and the Trimbos-institute

Because someone within a Recovery College often holds multiple roles, the terms "those involved" or "partakers" will generally be used.

2. Core values

This chapter describes several important core values that underpin the practice of Recovery Colleges. These core values appear throughout all other described core tasks or are related to them. Therefore, the core values are described first to lay the foundation for the model that follows. These core values are not scored because they are closely interconnected and difficult to assess in isolation. The description is based on the Professional Competency Profile of Lived Experience Expertise (van Erp and colleagues, 2023), the brochure 'Recovery Colleges' (Boertien and Harmsen, 2017), the WRAP Fidelity Scale (Lempens and colleagues, 2020), and the book 'Space for Peer Support!' (Muusse and Boumans, 2016).

Free space

A core value that underpins a Recovery College is free space. Free space primarily refers to inner space that a person can experience, even in complex or constraining circumstances, to (re)discover themselves, give meaning to one's life, make their own choices, and seek and find new possibilities. Experiencing this free space directly counters the disruption of what one is struggling with. Free space also represents the ability to exchange experiences freely without judgment or fixed frameworks (such as a medical-diagnostic framework or societal expectations). Finally, free space is also creating space for empowerment and influence of people who live in a disruptive situation, where this space is currently absent. The organizational free space that results from this must be actively and continuously safeguarded. This means that the Recovery College is not dictated top-down by entities such as a host organization, commissioner, or funder (e.g., a care organization or municipality), determining what occurs within the Recovery College and who is allowed to participate. The authority and ownership over the activities, policies, and decisions within the Recovery College always lies with those involved.

Recovery

In the 1990s, the concept of recovery was introduced by the user emancipatory within mental health care, leading to a shift in focus towards greater emphasis on meaningfulness and citizenship. Recovery is a unique developmental process in which individuals explore, based on their own experiences, what a meaningful life looks like to them. An important part of recovery is personal development, examining the 'sense of self': who am I, what can I do, and what do I want? Recovery also involves daring to look hopefully towards the future. It is not about curing, but about the (re-) development of skills, reconnecting with meaningful relationships, roles, and goals in life, with and beyond vulnerability. Recovery Colleges support these processes.

Empowerment

While recovery often focuses more on personal development, empowerment primarily addresses the relationship between the individual and society. Traditionally, empowerment has had an emancipatory meaning, related to fighting for space for people in vulnerable positions to influence their own lives. It is about strengthening the position of individuals so that they can take their rightful place in society. In addition to the individual aspect of empowerment – taking control of your life again – it is also a concept that involves a collective movement that can break through existing power structures and frameworks in society and care systems. Recognizing and utilizing experiential knowledge and expertise by experience play a crucial role in this. Within a Recovery College, all aspects of empowerment are present. A Recovery College provides space for partakers to choose what is suitable for their recovery at that moment (individual aspect). Additionally, a Recovery

© April 2024, Tranzo, Tilburg University and the Trimbos-institute

College pays necessary attention to destigmatization and the normalization of mental vulnerability (collective aspect).

Experiential knowledge and expertise by experience

Everything that someone knows and understands from lived experience (e.g., mental vulnerability, addiction, societal problems) is called experiential knowledge. By exchanging experiences in mutuality, collective experiential knowledge can also develop. Learning from the experiences of others contributes to better understanding one's own experiences and further developing one's own experiential knowledge. Experiential knowledge is a worthy source of knowledge alongside scientific and professional knowledge.

Expertise by experience is described as the ability of someone, based on both personal and collective experiential knowledge, to create space for others to discover and develop their own experiential knowledge. An expert by experience can share their own experiential knowledge but is not an expert on the experiences of others. Within a Recovery College, the exchange of experiences and experiential knowledge is an fundamental element: it shapes the collective learning environment.

Peer support

Peer support is about mutual, reciprocal (social/emotional) support from people with similar experiences (e.g., mental vulnerability). The relationships that peers build with each other are central in this; they offer an opportunity to share experiences, give and receive support, and build collective experiential knowledge that both parties can benefit from. Peer support focuses on hope and perspective for the future. However, this does not mean that difficulties and struggles cannot be discussed. Providing space to address struggles is precisely the strength of the peer support environment. It is about offering space for the struggles themselves and for experiences that encourage overcoming those struggles. A Recovery College facilitates such a peer support environment.

Anti-stigma

Recovery is not an isolated, individual process, but always takes place within a specific context or environment. Stigma is a negative label that influences how we see each other and ourselves. When such a label is assigned by ourselves, the care system, and/or society, it is called stigmatization. Stigmatization undermines recovery because it can limit the space for personal development. Sometimes this happens unconsciously, and sometimes it is explicitly woven into certain procedures, legislation, or rules. When people adopt certain stigmas as part of their own identity and believe in the negative stereotypes associated with them, it is called self-stigma. A key condition for recovery is breaking through (self-)stigma. A Recovery College is a place to address (self-)stigmatization and find societal activities to try to break and through (self-)stigma. This is necessary to increase societal opportunities, diversity, and inclusion, and to combat stigma and discrimination.

User emancipatory movement

Recovery Colleges originated from the user emancipatory movement in mental health care, which gained momentum in the 1970s. Clients saw mental health care as stigmatizing, paternalistic, and disempowering. The clients demanded control and decision-making over their lives and recovery processes. From this movement, beliefs emerged that working on recovery must be entirely voluntary, that individuals should be treated as equals, that recovery is possible, and that focusing on personal responsibility and strengths is essential. This considered, it is essential to maintain the independence and self-directed nature of Recovery Colleges, ensuring they remain “for and by” those involved.

© April 2024, Tranzo, Tilburg University and the Trimbos-institute

3. Core tasks – scoring instruction

This chapter describes ten core tasks of Recovery Colleges. These core tasks are scored in the online scoring form associated with this reflection tool[1]. The core tasks are visually represented below in a radar tool. The model is divided into four domains: personal domain, relational domain, collective domain, and organizational domain. Although the core tasks are not always exclusively prevalent in one domain but often span multiple domains, this classification can help making the layers of a Recovery College more tangible.

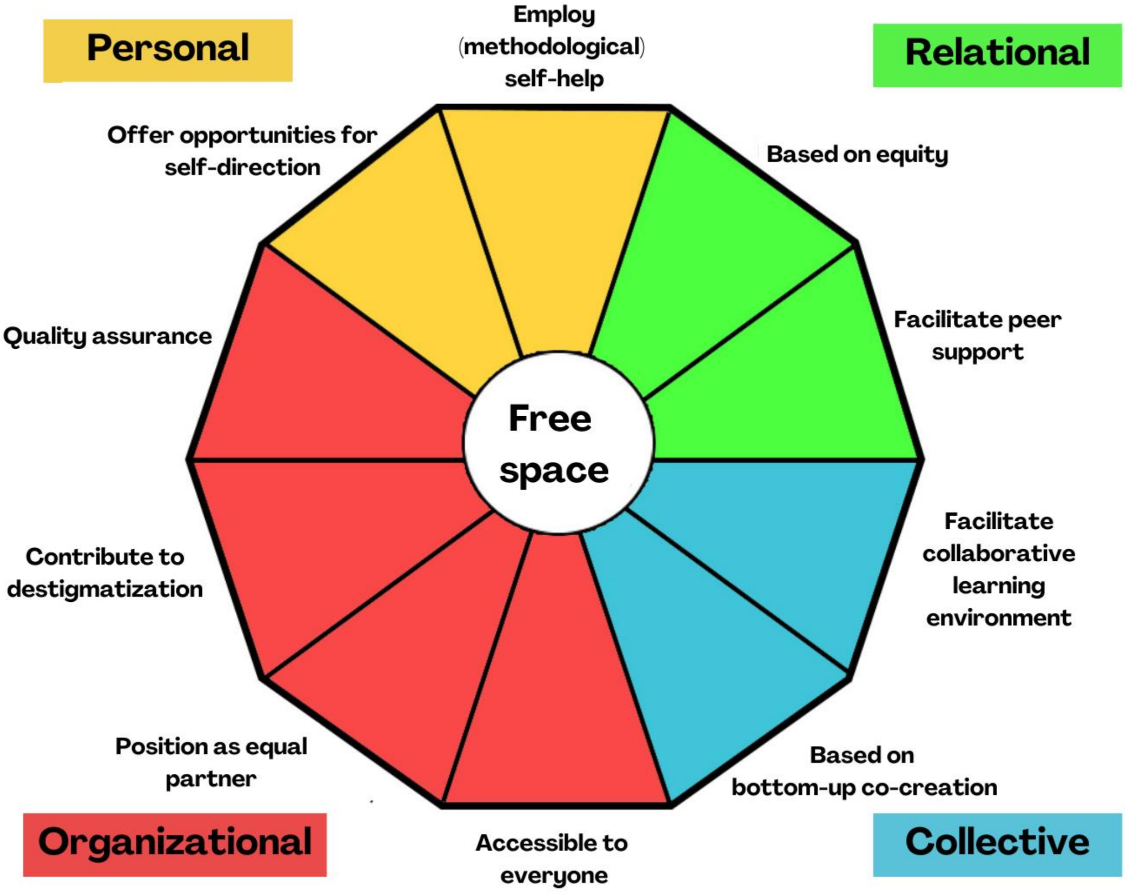

3.1. Personal domain

The personal domain is the layer of the experiences of participants, where the personal recovery process takes place.

Offer opportunities for self-direction

The Recovery College offers various opportunities for partakers to take more control over their lives and experience empowerment. Partakers are positioned to make their own choices about their lives again. They select activities from the program that best match their interests and needs at that moment. The focus on self-direction and empowerment also involves mutual trust so that one can

[1] This online scoring form was available for the testing phase of this tool in the Netherlands but is not publicly available at the moment.

© April 2024, Tranzo, Tilburg University and the Trimbos-institute

express their own needs and wishes and act on them. Ownership and personal responsibility are central.

To determine the extent to which your Recovery College offers sufficient opportunities for self-direction, you can consider the following:

- How many choices can someone make when they want to become active in the Recovery College?
  - Are the program offerings diverse enough in terms of **content**? For example, recovery working groups and activities on various topics such as finding meaning, coping with [...], peer work, personal meetings, lifestyle, well-being.
  - Are the program offerings diverse enough in terms of **intensity**? For example, one-time activities lasting a few hours, series of activities over several weeks, or events that take place over a day or half-day.
  - Are the program offers diverse enough in terms of **scheduling**? For example, activities in the morning, afternoon, evening, and spread throughout the year.
  - Are the program offers diverse enough in terms of **formats**? For example, creative methods, physical methods, cognitive methods, games, and theater.
  - Apart from the curricular program offerings, are there **other opportunities** within the Recovery College to develop oneself? For example, through different forms of volunteering.
  - Is there enough **freedom of choice** within the activities? For example, being able to give your own twist to an assignment, the freedom to skip certain parts, or not necessarily taking turns but having the option to speak or not during group discussions.
  - Is there enough **practical** freedom of choice? For example, the freedom to choose where to sit, or which color folder, paper, or pen to use.
- Does the Recovery College provide space for partakers to express their needs and act on them? For example, through a support document, as seen in Wellness Recovery Action Planning (WRAP).
- Does the Recovery College allow for an individual learning path? Does the Recovery College <u>not</u> use predefined curricula or processes that must be followed?

Employ (methodological) self-help

Methodological self-help is a form of support in which specific methods are used to explore the recovery process and personal experiences. The term “methodological” in this core task is in parentheses because methodological tools (such as Recovery is Up to You, Working with Own Experiences, WRAP, Honest Open Proud) can aid in facilitating self-help, but the emphasis is not on the use of those methods. The focus is on facilitating self-help as a process. The core of an environment where self-help is central is that facilitators provide space for participants to explore their own possibilities, desires, and needs. In this way, partakers learn how to influence the world around them and how to relate to it.

To determine the extent to which your Recovery College employs (methodological) self-help, you can consider the following:

- Are partakers encouraged to reflect on their strengths, capabilities, and qualities to gain insight into their possibilities?
- In what ways does the Recovery College facilitate this? For example, through recovery groups like WRAP or Recovery is Up to You, reflection exercises, writing your recovery story, as well as offerings outside the curriculum.

© April 2024, Tranzo, Tilburg University and the Trimbos-institute

3.2. Relational domain

The relational domain is the layer of underlying relationships and connections among the participants of the Recovery College.

Based on equity

All of those involved can contribute equally to the Recovery College. There is space for everyone's strengths and talents. Everyone is given equal opportunities to try things out and take on roles. People may have different roles and responsibilities (e.g., a location coordinator or a participant), but everyone remains equal as a person.

To determine the extent to which your Recovery College is based on equity, you can consider the following:

- Is it explicitly emphasized within the Recovery College that all experiences are of equal value? That no experience is better or worse than another?
    - Are facilitators taught how to ensure this in their activities?
    - Do facilitators explicitly state this at the start of an activity?
    - Does this come across in the (non)verbal communication among partakers, i.e., is there a non-judgmental attitude?
- Is the influence of roles and responsibilities on power dynamics regularly discussed? For example, the role of a location coordinator, a volunteer, or a participant.

Facilitate peer support

Within a Recovery College, peers are people with lived experience of mental vulnerability and recovery. By connecting with each other, partakers can give words to their experiences, find recognition and understanding, practice social contact, learn from each other, support one another, and build a social safety net. The Recovery College therefore facilitates an environment where peer support is central. This means that the Recovery College provides a safe space where people can be vulnerable and share their experiences (emphasizing the equality of all partakers). Within the Recovery College, reciprocity is essential: partakers not only learn to be supported by others but also to support others. This awareness can contribute to recovery.

To determine the extent to which your Recovery College facilitates peer support, you can consider the following:

- Does the Recovery College create a supportive environment where people provide each other with support through mutual connections?
    - Is this explicitly stated as an important pillar within the Recovery College?
- Within activities and recovery working groups, is there enough space and time for social contact besides the offered content? For example, icebreaker exercises and sufficient breaks.
- Is there space for lightness and humor within the Recovery College?
- Is there a space within the Recovery College where peers can meet other than during activities? For example, in a social meeting ground?
- Is the physical space designed to encourage social contact? Both in the rooms where activities are organized and in any potential meeting areas. For example, consider the arrangement of the space: an open room with round tables or square table arrangements that facilitate easy contact.

© April 2024, Tranzo, Tilburg University and the Trimbos-institute

3.3. Collective domain

The collective domain takes place at the level of the community, the collective, the whole that those involved in the Recovery College form together.

Facilitate a collaborative learning environment

The Recovery College facilitates a collaborative learning environment. Partakers work together, inspire each other, and learn from each other by sharing experiences, knowledge, and skills. Learning occurs not through the one-sided transfer of expertise or professional knowledge but through reciprocal and equal exchange. The Recovery College can also be seen as a space for practice.

To determine the extent to which your Recovery College facilitates a collaborative learning environment, you can consider the following:

- Is there enough time and space within activities and recovery working groups for partakers to exchange experiences and learn from each other? For example, in WRAP, the guideline is that 70% of the time in a recovery group should be reserved for exchange.
- Does the main program always take place in a group setting?
- In addition to "formal" learning opportunities (such as recovery groups and activities), does the Recovery College also provide space for informal learning opportunities (from social contact or taking on roles and responsibilities)?
- Are the physical spaces where recovery groups and activities take place designed as learning environments? For example, are whiteboards, flip charts, projectors, and/or workbooks used?
    - What does the rest of the Recovery College look like? Are there places available that are designed as learning environments? For example, a space with computers or a library with books on peer support and recovery.

Based on bottom-up co-creation

People with experiential knowledge (both facilitators and participants) shape the Recovery College. These can be professional experts by experience, but they do not have to be. Everything the Recovery College offers is developed bottom-up from the wishes, ideas, and needs of partakers. The Recovery College supports this process. This means that the Recovery College provides space for partakers to create activities based on their needs. Co-creation does not mean that every idea is immediately adopted, but rather that space is created to explore together how an idea can fit into the Recovery College.

To determine the extent to which your Recovery College is based on bottom-up co-creation, you can consider the following:

- Is the program developed based on the (wants and needs of) partakers themselves?
- Is there enough space for partakers to work out new ideas?
    - Is the process for developing an idea into a program component clear to everyone?
    - Are partakers regularly made aware of opportunities to develop their own ideas? A structured way to create space for co-creation could be by creating a pool where partakers with ideas are matched with facilitators focusing on specific themes.
- Are all activities within the Recovery College facilitated by at least one person with lived experience?
- Are people with lived experience in the lead?
- Is the physical space of the Recovery College co-created (and maintained) with the partakers?

© April 2024, Tranzo, Tilburg University and the Trimbos-institute

- During activities or recovery groups, is joint decision-making used where possible? For example, adjusting or skipping parts, deciding the timing of breaks, etc.
- To what extent do partakers have a say in how the Recovery College is given shape? For example, regarding policies, hiring staff, communication.
- If there is participation:
    - How is that participation facilitated? Does it take place in steering committee, recovery groups, etc.?
    - Is this participation structurally embedded or does it happen on an occasional basis?
    - Who makes the final decisions? Do the coordinators make these decisions, or do they make these decisions together with partakers?

3.4. Organizational domain

The organizational domain encompasses both the position of the Recovery College in society and the organizational aspects related to how the organization functions.

Accessible to everyone

The Recovery College is accessible to everyone (regardless of whether they are a client of a host organization, have a diagnosis, etc.). Although the Recovery College specifically targets people with mental vulnerabilities, anyone who feels drawn by the Recovery College is welcome. This means that the Recovery College strives for an open culture where everyone feels welcome and safe. No one is judged or treated differently because of their background or mental vulnerability. In principle, the Recovery College is a diagnosis-free space, meaning it doesn’t matter whether someone has diagnoses to begin with or which diagnoses they have. To ensure the Recovery College is accessible to everyone, attention is paid to low-threshold accessibility and diversity.

To determine how accessible your Recovery College is, consider the following:

- Is the Recovery College and are all recovery groups/activities accessible without a diagnosis? Or are there recovery groups or activities that only a limited group can participate in (e.g., clients of the host organization or people with a specific diagnosis)?
- Does the Recovery College offer a diagnosis-free space? In other words, does the Recovery College not ask for diagnoses?
- Are the physical locations easily accessible? For example, wheelchair accessibility, public transport availability, easy to find.
- Do the locations have a welcoming, warm atmosphere? Has attention been paid to the interior design to create a homely atmosphere (rather than a clinical one)?
- Is there an open culture within the Recovery College where everyone feels welcome and safe?
    - Have certain actions been taken to ensure this? For example, are shared behavioral agreements or core values clearly visible to everyone?
- Are the activities financially accessible? (Compare: one can attend for free vs. a financial contribution is expected)
- Is the report on someone’s process always in the hands of the individual themselves?
    - Are <u>no</u> internal records kept for, for example, progress monitoring of partakers?
    - Are <u>no</u> personal details (e.g., name, contact details, address, which programs someone has attended) shared with third parties (e.g., funders, municipalities, host organizations)?

© April 2024, Tranzo, Tilburg University and the Trimbos-institute

- Are individual learning needs of partakers considered? For example, printing workbooks with larger text for people with dyslexia, or translating content into another language for participants who do not speak the primary language.

Position as equal partner

The Recovery College ensures visibility in the neighborhood and region. The Recovery College aims not only to encourage participants to take meaningful roles within the Recovery College but also outside of it. The Recovery College is aware of the balance it must maintain between facilitating a safe peer support environment and collaborating with other (care) organizations. The Recovery College can leverage its expertise in peer support and self-direction to support or advise other organizations. The Recovery College also aims to influence policy within a broader network.

To determine how well your Recovery College is positioned as an equal partner, consider the following:

- Is the Recovery College active in the neighborhood/region to raise awareness of their offerings?
    - In what ways is this done? For example, organizing external information sessions, distributing flyers and informational brochures, participating in relevant networks.
    - How often is this done?
    - Is it done structurally or occasionally?
- Does the Recovery College consider how the facilitated peer support environment can integrate with the neighborhood/region? In other words, does it encourage an open attitude toward the community to prevent the Recovery College from becoming too inward-focused?
    - How is this done? For example, through team meetings, discussions with neighborhood representatives, brainstorm sessions with partakers.
- Does the Recovery College collaborate with relevant partners in the neighborhood/region? For example, welfare organizations, community centers, sports clubs, cultural associations, schools.
    - Does the Recovery College offer flyers or informational brochures from relevant partners to help partakers find other activities in the neighborhood/region?
    - Are joint events or activities offered to help partakers find their way to other activities in the neighborhood/region?
- Are partakers in the Recovery College actively informed about opportunities in the neighborhood/region, or for example are there merely flyers presented at a reception desk?
- Does the Recovery College share knowledge and expertise about peer support and self-direction with relevant partners in the neighborhood/region?
- If the Recovery College collaborates with partners in the neighborhood/region: is the Recovery College's identity actively maintained in this collaboration? For example, through adherence to core values, a consistent brand style, etc.
    - How is this done?
    - Is this always successful, or sometimes not?

Contribute to destigmatization

A Recovery College plays an important role in reducing stigma and discrimination, and promoting inclusion. This concerns destigmatization at all levels: breaking down self-stigma, stigma in care, and stigma in society. Attention is not only given to the impact of stigmatization, but also to becoming

© April 2024, Tranzo, Tilburg University and the Trimbos-institute

aware of one’s own stigmatizing behavior. It is also about creating space for plurality of voices. This space should be facilitated within the Recovery College, but also outside in society. The goal is to normalize variation and diversity. Contributing to destigmatization can also be achieved by, for example, actively advocating or working as a pioneer in the neighborhood/society to strengthen the position of people with mental vulnerabilities.

To determine how much your Recovery College contributes to destigmatization, consider the following:

- Does the Recovery College pay significant attention to the themes of self-stigma, stigma, and destigmatization?
  - How is this reflected in programs they offer, for example?
- Is there regular discussion about the presence and impact of stigma within the Recovery College?
- Is the Recovery College actively working to combat stigma in healthcare?
  - How is this done? For example, through meetings with healthcare providers, organizing lectures or workshops on this topic for healthcare providers.
- Is the Recovery College actively working to combat stigma in society?
  - How is this done? For example, through advocacy, organizing lectures or workshops on this topic in the neighborhood or even beyond.

Quality assurance

The Recovery College is intensively and structurally engaged in ensuring the quality of the program offerings. Ideally, these processes not only take place within the Recovery College but also in exchange between different Recovery Colleges. During these processes, reflecting on the core values in practice is central. Dilemmas can thus be discussed, and valuable lessons can be learned.

To determine how much your Recovery College is engaged in quality assurance, consider the following:

- Are meetings organized within the Recovery College in the form of peer consultation, supervision, subject groups, or ethical deliberations?
  - Are these meetings organized regularly, or only incidentally (i.e., when there is a direct occasion)?
  - Are these meetings organized internally only, or is there also an exchange with other Recovery Colleges (e.g., in the region)?
- Has the role of ‘facilitator’ been clearly described within the Recovery College, for example, in a job description?
- Is there enough time, space, and resources for facilitators to continue their professional development? For example, by offering the WRAP refresher training or staying up to date with (scientific) literature?
- Does the Recovery College collaborate with relevant educational institutions (such as those offering training for peer specialists) to keep knowledge and skills up-to-date? For example, by offering internship placements?
- Is there a plan for the further development of the Recovery College described (in co-creation)?
  - And is this regularly reviewed?

© April 2024, Tranzo, Tilburg University and the Trimbos-institute

4. Practical choices

In this chapter, several practical choices are presented. One choice is not better or worse than another. It can provide insight into possible forms of a Recovery College. Sometimes, italicized explanations or reflections are provided alongside the practical choices.

Program offerings

Facilitating a peer support learning environment can be done through various offerings. Offering recovery-oriented series, activities, and recovery working groups is an essential part of a Recovery College. In addition, a Recovery College can provide other offerings. What types of offerings does your Recovery College provide?

☐ (Recovery-oriented) series, activities and recovery work groups

☐ Social meeting ground

☐ Volunteer opportunities

☐ Retreats (multi-day series including overnight stays)

☐ Other, namely ____

Program scope

Within a Recovery College, exchanging about and reflecting on recovery is central. Therefore, the program focusses on exploring one's own recovery (story) in exchange with peers. However, one can also choose to offer a program with a broader scope. For example, a Recovery College might offer working groups or activities that are also offered by other initiatives (e.g., community centers, gyms). Think of activities like yoga, walking, cooking together, photography. These types of activities can be used to stimulate reflection on recovery (e.g., exchanging thoughts on a recovery theme during a walk), but they can also be offered without that focus (e.g., walking together for fun). What is the scope of your Recovery College?

A = (**Almost) none** of our offerings is specifically focused on exploring and exchanging about one's own recovery (story). Our program almost always has a broader scope.

B = A **small part** of our offerings is specifically focused on exploring and exchanging about one's own recovery (story). The largest part of our program has a broader scope.

C = **About half** of our offerings is specifically focused on exploring and exchanging about one's own recovery (story). The other half of our program has a broader scope.

D = The **largest part** of our offerings is specifically focused on exploring and exchanging about one's own recovery (story). A small part of our program has a broader scope.

E= **(Almost) all** of our offerings is specifically focused on exploring and exchanging about one's own recovery (story).

Co-creation partnerships

Co-creation within a Recovery College always involves people with lived experience (peers). Activities can be designed and facilitated solely by peers, or peers can collaborate with a healthcare provider, expert, or external professional.

© April 2024, Tranzo, Tilburg University and the Trimbos-institute

Note: Healthcare providers, experts, or other external professionals can sometimes also be peers. When they contribute from their lived experience in exchange, they are considered peers.

Note: An activity must never be designed and facilitated only by healthcare providers/experts/external professionals. Co-creation must always be initiated by peers/experts with lived experience, and they must be involved throughout the entire process. When co-creation with experts takes place, special attention must be given to maintaining equity and reciprocity (exchanging experiences, not training based on expertise).

What partnerships exist within your Recovery College?

A = **(Almost) all** of our offerings is designed and facilitated by peers **together** with a healthcare provider, expert or external professional.

B = The **largest part** of our offerings is designed and facilitated by peers **together** with a healthcare provider, expert or external professional. A small part of our program is designed and facilitated **solely by peers**.

C = **About half** of our offerings is designed and facilitated by peers **together** with a healthcare provider, expert or external professional, the other half is designed and facilitated **solely by peers**.

D = The **largest part** of our offerings is designed and facilitated **solely by peers**. A small part of our program is designed and facilitated **together** with a healthcare provider, expert or external professional.

E = **(Almost) all** of our offerings is designed and facilitated **solely by peers**.

Type of location

A Recovery College can be housed in different types of locations. What type(s) of location(s) apply to your Recovery College?

Note: If your Recovery College only has one location, provide the answer for that specific location. If your Recovery College has multiple locations, include all locations in your answer.

Note: An "own location" means that the location is not shared with any other organization, and the building is managed by the Recovery College or the host organization.

Note: A "shared location" refers to buildings shared with other social or public services (such as a community organization, community center, library) or buildings from a mental health organization (i.e., a building where treatment/support also takes place).

A = (**Almost) all** of our locations are shared with other organizations.

B = The **largest part** of our locations is shared with other organizations. A small part of our locations is housed in owned buildings.

C = **About half** of our locations is shared with other organizations, the other half is housed in owned buildings.

D = The **largest part** of our locations are own locations. A small part of our locations is housed in locations shard with other organizations.

© April 2024, Tranzo, Tilburg University and the Trimbos-institute

E = **(Almost) all** of our locations are own locations.

Reflections

Having an own building can be empowering, and as an organization, you have full control over shaping your space. This way, the identity of the Recovery College can be strongly established. Additionally, owning a building supports the creation of a peer support community.

Using a shared location can be more accessible and make it easier for people to find the Recovery College. However, it is crucial to protect the identity of the Recovery College and to ensure sufficient influence in shaping its space. A location should always aim to clearly communicate its own identity, for example, by having its own entrance, a dedicated reception area, or branding on the windows.

One or more main locations owned by the Recovery College, combined with offering activities in the community, can complement each other. Low-threshold activities in the community can spark partakers' curiosity to visit the main location(s) as well.

A location that shares a building where mental health services are also offered may conflict with the core values of a Recovery College. Independence from mental health services is important to emphasize the position of the Recovery College and to continue creating space for the described core values and core tasks.

© April 2024, Tranzo, Tilburg University and the Trimbos-institute

5. Bibliography

To formulate the core values and core tasks, information was drawn from:

- Focus groups with 16 Recovery Colleges in the Netherlands (30 participants → facilitators and coordinators)
- Exchanges with the POP Group (experiential co-researchers from Enik Recovery College, Utrecht, the Netherlands)
- Exchanges with experts from MIND, the Dutch Association for Self-Direction and Recovery, and Cavallo Advice (part of the 'Blauwe Paard Netwerk')

And the following references:

Boertien, D., & Harmsen, K. (2017). *Herstelacademie - vrijplaats voor eigen ontwikkeling*. Utrecht: Werkplaats Herstelondersteuning. https://kenniscentrumphrenos.nl/document/herstelacademie-vrijplaats-voor-eigen-ontwikkeling/

Hellweg, K. (2020). *Bouwstenen voor participatie en herstel.* MIND Landelijk Platform Psychische Gezondheid. https://mindplatform.nl/media/4854/download/MIND%20Bouwstenen%20voor%20participatie%20en%20herstel_def.pdf?v=1

Lempens, A., de Lange, A., Boertien, D., & van Bakel, M. (2021). *Modelgetrouwheidsschaal WRAP.* Utrecht: Kenniscentrum Phrenos en Trimbos-instituut. https://www.trimbos.nl/aanbod/webwinkel/af1887-modelgetrouwheidsschaal-wrap/

Muusse, C. & Boumans, J. (2016). *Ruimte voor Peer Support! Een onderzoek naar de totstandkoming van Enik Recovery College.* Utrecht: Lister. https://www.trimbos.nl/actueel/nieuws/ruimte-voor-peer-support/

Nederlandse Vereniging voor Zelfregie en Herstel (2022). *Criteria om lid te worden van de NVZH.* https://nvzh.nl/wp-content/uploads/2022/02/NVZH_Criteria_lidmaatschap.pdf

Toney, R., Knight, J., Hamill, K., Taylor, A., Henderson, C., Crowther, A., Meddings, S., Barbic, S., Jennings, H., Pollock, K., Bates, P., Repper, J., & Slade, M. (2019). Development and Evaluation of a Recovery College Fidelity Measure. *The Canadian Journal of Psychiatry, 64*(6), 405-414. https://doi.org/10.1177/0706743718815893

van Erp, N., Boertien, D., Liefhebber, S., & van den Berg, A. (2022). *Beroepscompetentieprofiel Ervaringsdeskundigheid.* Utrecht: Kenniscentrum Phrenos. https://kenniscentrumphrenos.nl/kennisproduct/beroepscompetentieprofiel-ervaringsdeskundigheid/

Workgroup accessible support centers. (2023). *Landelijk dekkend netwerk van laagdrempelige steunpunten - Handvatten voor de regio's in het kader van realisatie van de IZA-afspraak over laagdrempelige steunpunten*.

© April 2024, Tranzo, Tilburg University and the Trimbos-institute

6. Acknowledgments

Authors:

Marloes van Wezel, Christien Muusse, Dike van de Mheen, Hans Kroon

Design:

Kelly Leunen

Translation:

Kelly Leunen, Marloes van Wezel

Technical support:

Mark Vergeer

Thanks to:

- Jenny Boumans, Annelies Broos, Judith Lizé, René van de Male, Gaby Dekkers, Karin Goudsmit, Katinka Hellweg, Tim Kreuger, Martijn Kole, Willeke Keyman, Roel Suidgeest, Greetje Senhorst, Sonja Visser, Elske van der Velde, Ton Verspoor, Joes Adriaansens, Michiel Bahler, Manuela Blaswich, Fieke Bosscher, Chantal de Bont-Melkstop, Samirah Daniëls, Daniejel de Greef, Gerard de Roos, Olga de Vogel, Daphne Doorn, Angela van Dongen, Lori van Egmond, Marjolein Jacobs-Orsel, Gert Jansen, Margreet Kingma, Martijn Koopmans, Marcia Kroes, Marlous Langeland, Sarah Lewis, Rosa van Mourik, Martijn Nouwels, Marco Smit, Chantal Soepboer, Eva Velthorst, Menno Veenstra, Karianne Vogel, Nanette Waterhout, Mickal Weggelaar, Josse Weyers, Dick Zwiers for their input and advice.
- Ewout Kattouw, Wout Diekman, Floris Scheerstra, Hannah Holleman, Kelly Leunen for their support during data collection.

© April 2024, Tranzo, Tilburg University and the Trimbos-institute

7. Attachment – template radar tool

The filled-out online reflection tool leads to a dashboard where the scores on the core tasks are visually represented with bar graphs. The practical choices are also visually displayed there.

In the model description of this document, we used a radar tool visualization. A blank version of such a radar tool is shown on the next page. The scores can be marked here if desired, to create your own radar tool.

For example, imagine these are the scores for your Recovery College on the core tasks:

- Offer opportunities for self-direction: 4
- Employ (methodological) self-help: 3
- Based on equity: 4
- Facilitate peer support: 3
- Facilitate collaborative learning environment: 2
- Based on bottom-up co-creation: 2
- Accessible to everyone: 3
- Position as equal partner: 4
- Contribute to destigmatization: 3
- Quality assurance: 2

Then the radar tool would look like this:

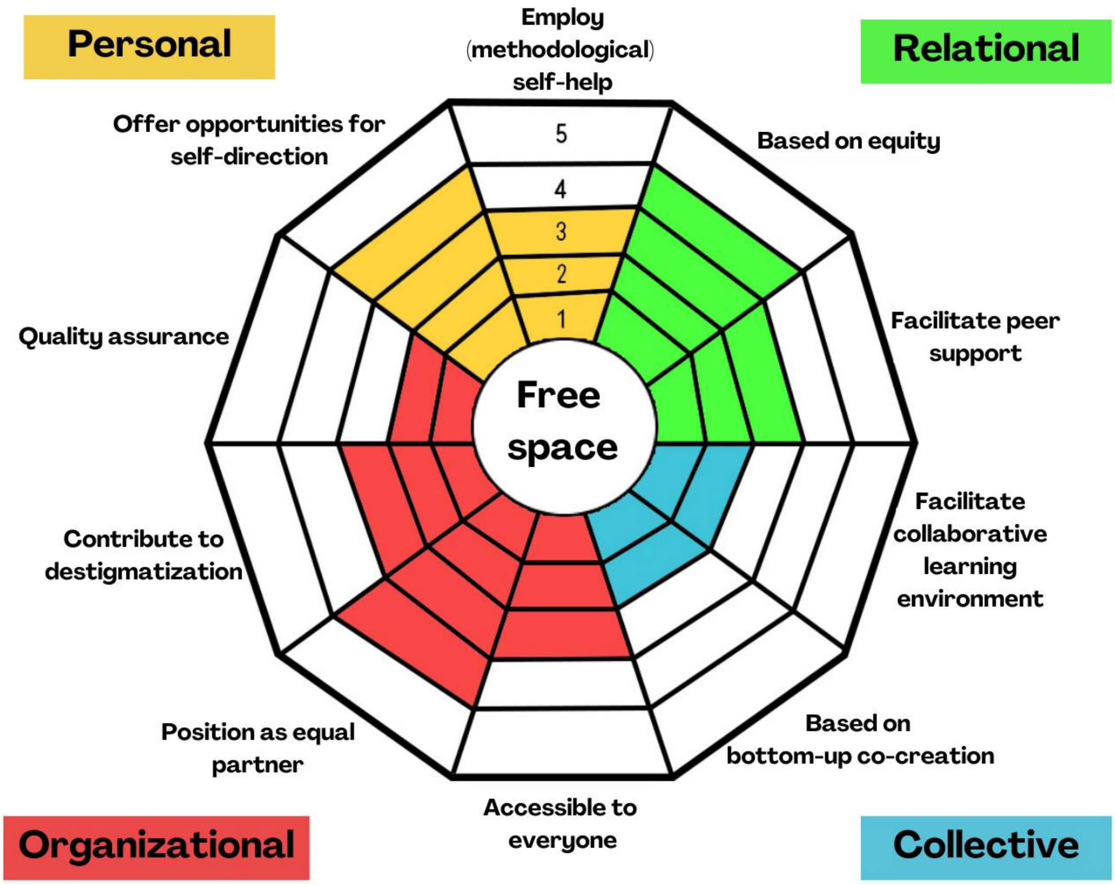

If desired, enter the scores for the core tasks for your Recovery College here:

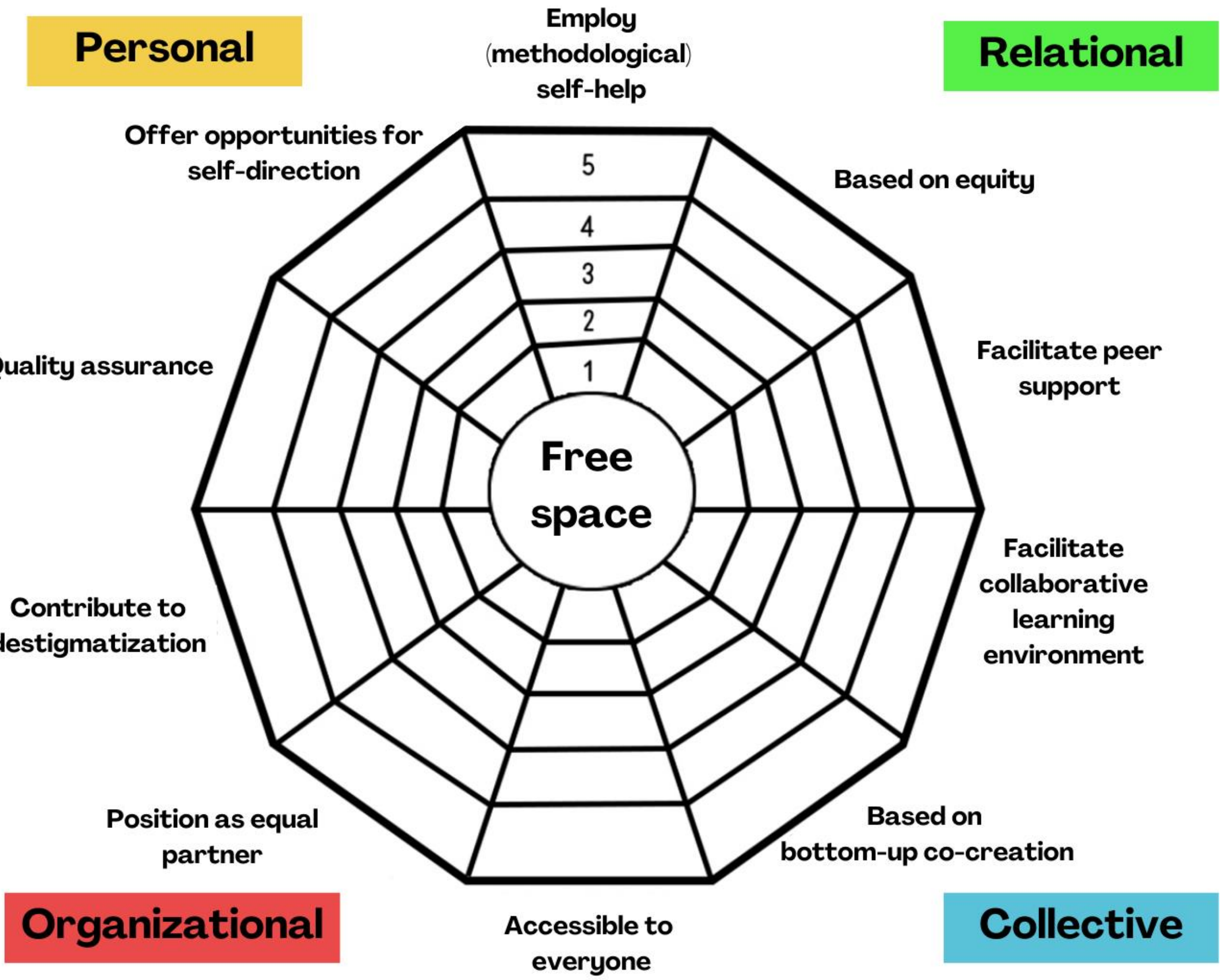

© April 2024, Tranzo, Tilburg University and the Trimbos-institute

Appendix D

Bar plots of core tasks and practical choices of Dutch RCs

Figure D.1

Bar plots of the ten core tasks as specified in the reflection tool (N= 24)

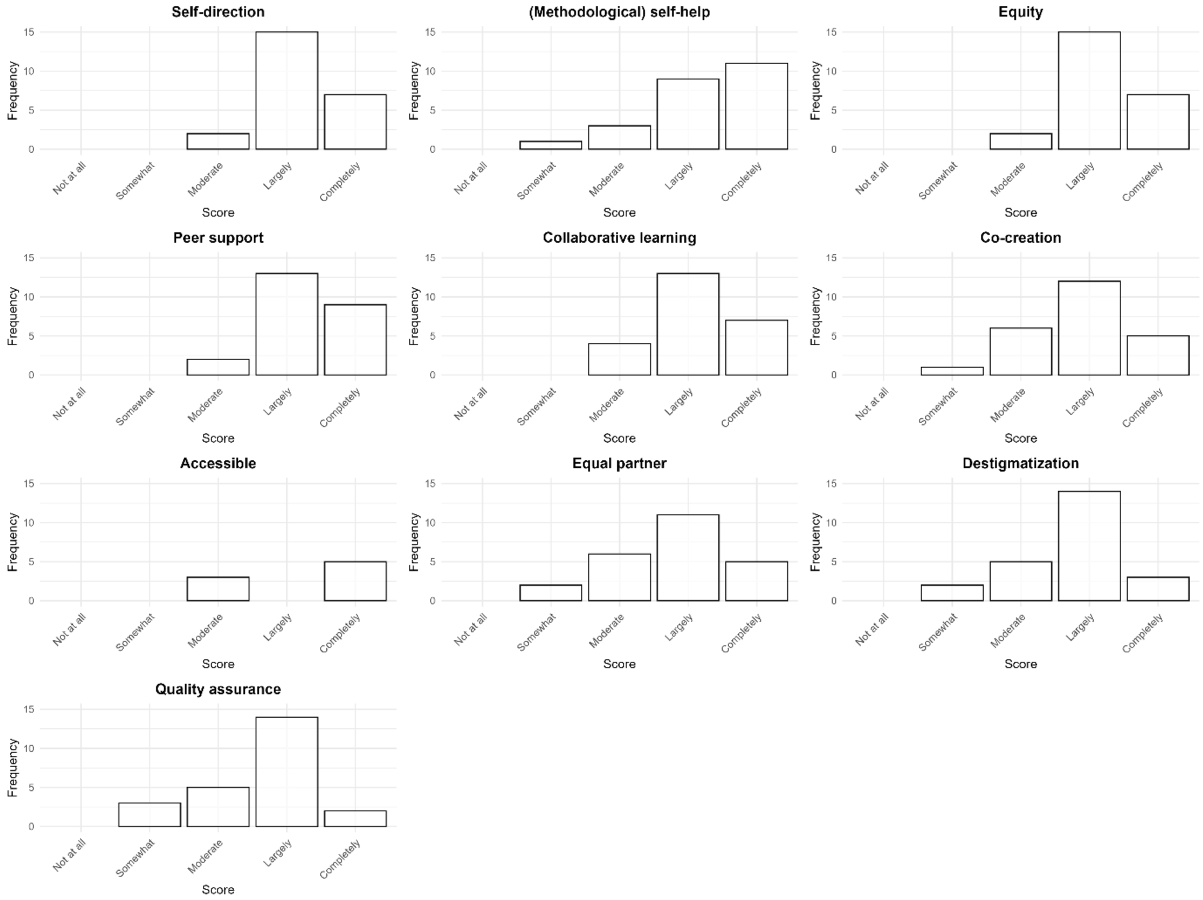

Figure D.2

Bar plots of the three practical choices as specified in the reflection tool (N = 24)

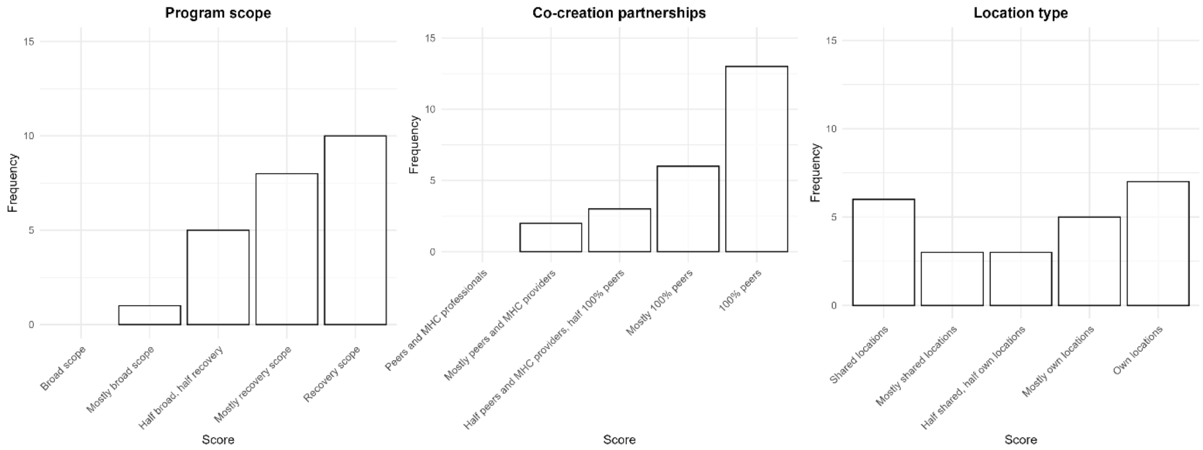

Supplement: Supplementary file 1 — (PDF 1.43 MB) [file 10597_2025_1517_MOESM1_ESM.pdf]
